# European Headache Federation (EHF) critical reappraisal and meta-analysis of oral drugs in migraine prevention – part 3: topiramate

**DOI:** 10.1186/s10194-023-01671-5

**Published:** 2023-10-10

**Authors:** Bianca Raffaelli, David García-Azorín, Deirdre M. Boucherie, Faisal Mohammad Amin, Christina I. Deligianni, Raquel Gil-Gouveia, Sarah Kirsh, Christian Lampl, Simona Sacco, Derya Uluduz, Jan Versijpt, Antoinette MaassenVanDenBrink, Dena Zeraatkar, Margarita Sanchez-del-Rio, Uwe Reuter

**Affiliations:** 1 Department of Neurology, Charité Universitätsmedizin Berlin, Berlin, Germany; 2 Clinician Scientist Program, Berlin Institute of Health at Charité (BIH), Berlin, Germany; 3 Headache Unit, Neurology Department, Hospital Clínico Universitario de Valladolid, Valladolid, Spain; 4 Department of Internal Medicine, Division of Vascular Medicine and Pharmacology, Erasmus MC Medical Center, Rotterdam, the Netherlands; 5 Department of Neurology, Danish Headache Center, Copenhagen University Hospital - Rigshospitalet, Copenhagen, Denmark; 6 Department of Brain and Spinal Cord Injury, Copenhagen University Hospital – Rigshospitalet, Copenhagen, Denmark; 7 Department of Neurology, Athens Naval Hospital, Athens, Greece; 8 Hospital da Luz Headache Center, Neurology Department, Hospital da Luz Lisboa, Lisbon, Portugal; 9 Center for Interdisciplinary Research in Health, Universidade Católica Portuguesa, Lisbon, Portugal; 10 Department of Anesthesia and Department of Health Research Methods, Evidence and Impact, McMaster University, Hamilton, Canada; 11 Department of Neurology and Stroke Unit, Konventhospital Barmherzige Brüder Linz, Linz, Austria; 12 Headache Medical Center Linz, Linz, Austria; 13 Department of Biotechnological and Applied Clinical Sciences, University of L’Aquila, L’Aquila, Italy; 14 Department of Neurology Istanbul Cerrahpasa Medical Faculty, Istanbul, Turkey; 15 Department of Neurology, Vrije Universiteit Brussel (VUB), Universitair Ziekenhuis Brussel (UZ Brussel), Brussels, Belgium; 16 Department of Neurology, Clinica Universidad de Navarra, Madrid, Spain; 17 Universitätsmedizin Greifswald, Greifswald, Germany

## Abstract

**Objective:**

Topiramate is a repurposed first-line treatment for migraine prophylaxis. The aim of this systematic review and meta-analysis is to critically re-appraise the existing evidence supporting the efficacy and tolerability of topiramate.

**Methods:**

A systematic search in MEDLINE, EMBASE, Cochrane CENTRAL, and ClinicalTrials.gov was performed for trials of pharmacological treatment in migraine prophylaxis as of August 13, 2022, following the Preferred Reporting Items for Systematic Reviews (PRISMA). Randomized controlled trials in adult patients that used topiramate for the prophylactic treatment of migraine, with placebo as active comparator, were included. Two reviewers independently screened the retrieved studies and extracted all data. Outcomes of interest were the 50% responder rates, the reduction in monthly migraine days, and adverse events leading to treatment discontinuation. Results were pooled and meta-analyzed, with sensitivity analysis based on the risk of bias of the studies, the monthly migraine days at baseline, and the previous use of other prophylactic treatments. Certainty evidence was judged according to the GRADE framework.

**Results:**

Eight out of 10,826 studies fulfilled the inclusion/exclusion criteria, accounting for 2,610 randomized patients. Six studies included patients with episodic migraine and two with chronic migraine. Topiramate dose ranged from 50 to 200 mg/day, and all studies included a placebo arm. There was a high certainty that topiramate: 1) increased the proportion of patients who achieved a 50% responder rate in monthly migraine days, compared to placebo [relative risk: 1.61 (95% confidence interval (CI): 1.29–2.01); absolute risk difference: 168 more per 1,000 (95% CI: 80 to 278 more)]; 2) was associated with 0.99 (95% CI: 1.41–0.58) fewer migraine days than placebo; 3) and had a higher proportion of patients with adverse events leading to treatment discontinuation [absolute risk difference 80 patients more per 1,000 (95% CI: 20 to 140 more patients)].

**Conclusions:**

There is high-quality evidence of the efficacy of topiramate in the prophylaxis of migraine, albeit its use poses a risk of adverse events that may lead to treatment discontinuation, with a negative effect on patient satisfaction and adherence to care.

**Supplementary Information:**

The online version contains supplementary material available at 10.1186/s10194-023-01671-5.

## Introduction

In the late 1970s, a series of novel anticonvulsant class drugs, O-alkyl sulfamates, were developed. Out of 26 essayed drugs, McN-4853, also known as [2,3:4,5-bis-O-(1-methylethylidene-beta-D-fructopyranose sulfate], or ultimately as topiramate, showed an adequate balance of preclinical effectivity and minimal neurotoxicity, and it was selected for clinical development as a novel anticonvulsant drug [1]. In migraine, the first experiences date from the late 1990s as case series [2–4]. These promising results were followed by the first double-blind, placebo-controlled, randomized controlled trials, providing the necessary evidence [5, 6] for its label as a migraine prophylactic drug and its inclusion in the guidelines for migraine treatment [7].

**Fig. 1:**
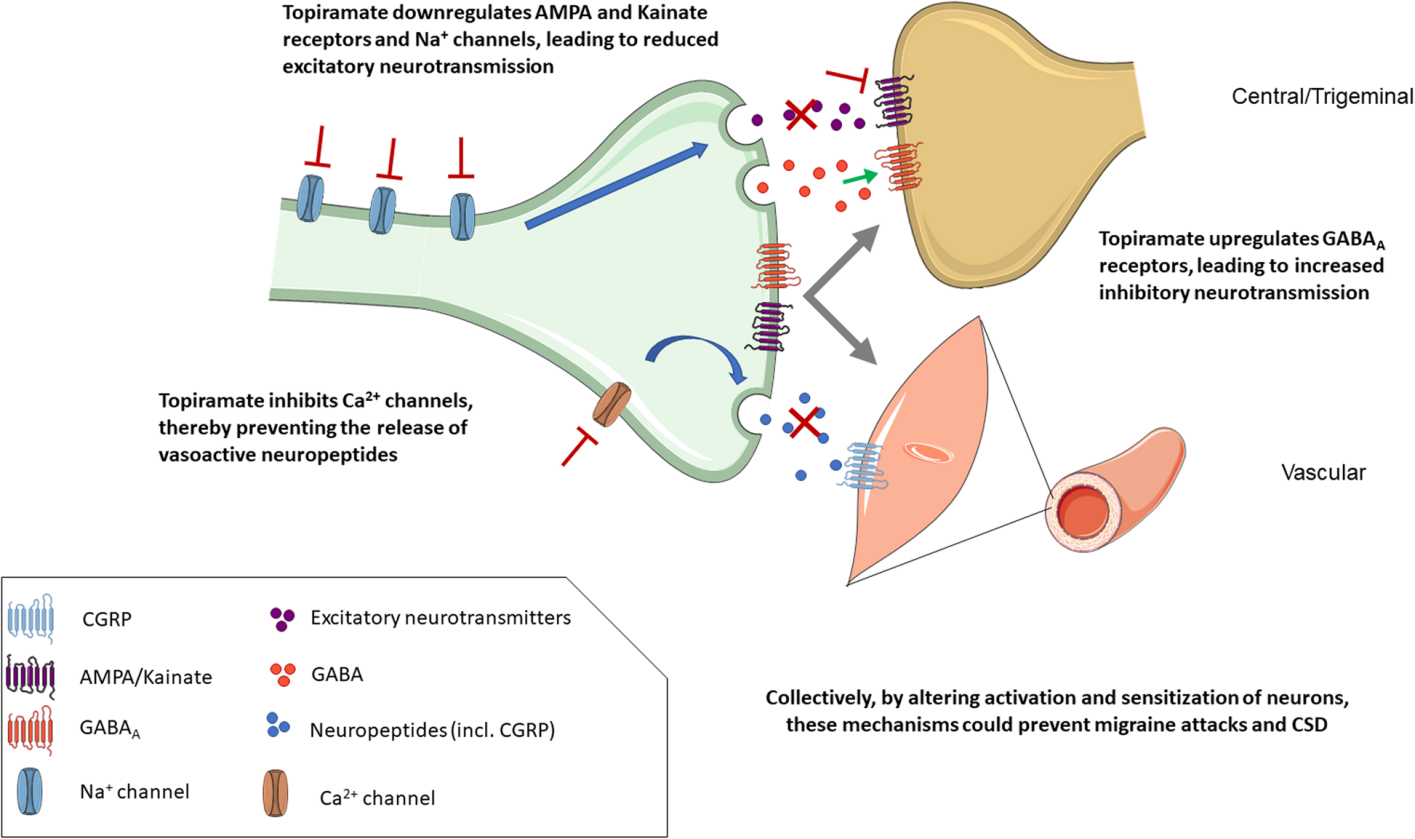
Potential mechanisms of action for the anti-migraine effect of topiramate. Topiramate is proposed to act via inhibition of nociceptive neuronal firing in the trigeminocervical complex in animal models [12]. Firstly, excitatory neurotransmission is reduced via downregulation of postsynaptic AMPA/kainate receptors and Na + channels [13–15]. Moreover, topiramate enhances GABA_A_ receptor activity, thereby promoting inhibitory GABAergic neurotransmission [14, 15]. By inhibition of voltage-gated Ca^2+^ channels, topiramate may prevent the release of vasoactive neuropeptides including CGRP [16]. Collectively, by altering activation and sensitization of neurons, these mechanisms could prevent migraine attacks and cortical spreading depression [8, 17]

Anticonvulsants are commonly used for the prophylaxis of migraine and some targets shared by this class include inhibition of the pathways involved in excitatory neurotransmission by glutamate, stimulation of inhibitory neurotransmission by γ-aminobutyric acid (GABA), as well as negative modulation of voltage-gated Na^+^ and Ca^2+^ channels, leading to decreased neuronal excitability [8–11]. The exact mechanisms which account for the prophylactic effect of topiramate in migraine remain unknown. It is proposed to act via inhibition of nociceptive neuronal firing in the trigeminocervical complex in animal models [12]. Topiramate can bind to AMPA and kainate receptors as well as Na^+^ channels in a dephosphorylated state, leading to downregulation of these targets [13]. As such, it can reduce the release of excitatory neurotransmitters [14, 15]. Moreover, topiramate is suggested to prevent the release of vasoactive peptides including calcitonin gene-related peptide (CGRP) by inhibition of voltage-gated Ca^2+^ channels on trigeminal nerve endings [16]. Upon binding to the GABA_A_ receptors on neurons, topiramate may enhance the activity of GABA_A_, thereby promoting inhibitory GABA neurotransmission [13–15]. Lastly, chronic administration of topiramate was demonstrated to suppress cortical spreading depression in a rat model, which is thought to be implicated in the pathophysiology of migraine aura [17]. Topiramate is also a weak carbonic anhydrase inhibitor [1]. Figure 1 summarizes the mechanism of action of topiramate.

Topiramate is considered as a first-choice drug for migraine prophylaxis. The aim of this study was to conduct a critical reappraisal of the existing evidence supporting the efficacy and tolerability of topiramate as a treatment for migraine prophylaxis.

## Methods

This study is the third manuscript of a series of critical reappraisals of migraine prophylactic drugs. Methods are reported in detail in the preceding publications [18, 19]. In the following paragraphs, the key methodological aspects will be briefly mentioned.

### Study design

To evaluate the efficacy and tolerability of topiramate in the preventive treatment of migraine, a systematic review and meta-analysis were conducted. The results are reported according to the Preferred Reporting Items for Systematic reviews and Meta-Analyses (PRISMA) statement, 2020 version [20].

### Eligibility criteria

The inclusion criteria were: 1) randomized controlled trials; 2) trials in which 80% or more of patients are older than 18 years; 3) diagnosis of episodic or chronic migraine, according to established criteria; 4) focused on the prophylactic treatment of migraine; 5) having topiramate as one of the interventions; 6) with placebo as active comparator.

Studies were excluded if: 1) they were systematic reviews, scoping reviews, or narrative reviews; 2) the study design was non-randomized (i.e. cohort studies, case–control studies, or cross-sectional studies); 3) the study was focused on acute treatment; 4) the number of study participants per treatment arm was < 25; 5) the results were reported only as conference abstracts.

### Information sources

A literature search in MEDLINE, EMBASE, Cochrane CENTRAL and ClinicalTrials.gov databases was conducted, looking for trials of topiramate in migraine prevention.

### Search strategy

The search included the time span between database inception and August 13, 2022, without language restrictions. In addition, we searched for relevant literature in the bibliographies of other systematic reviews and meta-analyses about topiramate [21]. Supplementary Material 1 presents the full search strategy.

### Selection process

Following training and calibration exercises to ensure sufficient agreement, two reviewers worked independently. First, titles and abstracts of search records were screened, and subsequently, the full text of records that deemed eligible were evaluated in detail.

### Data collection process

Two independent reviewers collected all the study variables and completed a pre-defined database.

### Data items – outcomes of interest

From the included trials, the following outcomes of interest were extracted: 50% responder rates, i.e. proportion of patients who experience a 50% or more reduction in monthly migraine days during the treatment period, when compared with the baseline period; monthly migraine days; and adverse events leading to treatment discontinuation [22, 23]. In case monthly migraine days were not reported, we used the number of monthly migraine attacks as surrogate parameter for all outcomes of interest. Outcomes were selected based on the International Headache Society guidelines for controlled trials of preventive treatment of migraine in adults, and were harmonized in all the studies of these EHF series [18, 19]. Data was extracted at the latest reported follow-up point in which patients were still receiving topiramate.

Trials reported on a range of doses of topiramate from 50 to 200 mg, with most trials reporting on 100 mg. In our primary analysis, we combine all doses of topiramate, since most data comes from trials reporting on 100 mg and based on sensitivity analyses that suggested excluding studies with doses less than 100 mg had minimal impact on the pooled effect estimate. When trials compared more than one dose of topiramate with placebo, we combined the results of all doses together using methods described by Rucker and colleagues [24].

A series of additional variables were collected, including the trial name, the specific intervention, the trial duration, the number of randomized patients, the type of migraine (episodic vs. chronic), the monthly migraine days per month at baseline, gender, and age.

### Study risk of bias assessment

For each trial, two independent reviewers evaluated the risk of bias using a modified Cochrane RoB 2.0 tool [25, 26]. Several domains were considered, including the randomization process, deviations from the intended intervention, missing outcome data, outcome measurements, and selection of reported results. Each domain was rated as either “low risk of bias”, “some concerns—probably low risk of bias”, “some concerns—probably high risk of bias”, or “high risk of bias”.

### Synthesis methods

First, a narrative summary of the evidence was performed. Second, the study results were pooled and meta-analyzed. The meta-analyses for all outcomes were performed using the *meta* and *metafor* packages in R (version 4.1.2) [27, 28], as previously described [18]. In addition, sensitivity analyses were performed by subgroup pairwise meta-analysis and meta-regressions based on the following trial characteristics: risk of bias (low vs. high); monthly migraine days at baseline (below vs. above the median value); participants with previous use of other prophylactic treatments.

Variability between studies was assessed with the τ^2^ between study variance, and study heterogeneity was estimated with the I^2^ statistic, considering an I^2^ > 50% as a statistically significant heterogeneity.

### Certainty of evidence assessment

Using the GRADE approach [29], the certainty of evidence was evaluated, by assessing the risk of bias, inconsistency, indirectness, imprecision, and publication bias for each comparison. The quality of each comparison was rated as either high, moderate, low, or very low. We applied GRADE simple language summaries to report the results, using declarative statements for high certainty evidence, “probably” for moderate certainty evidence, “may” for low certainty evidence, and “very uncertain” for very low certainty evidence.

## Results

**Fig. 2:**
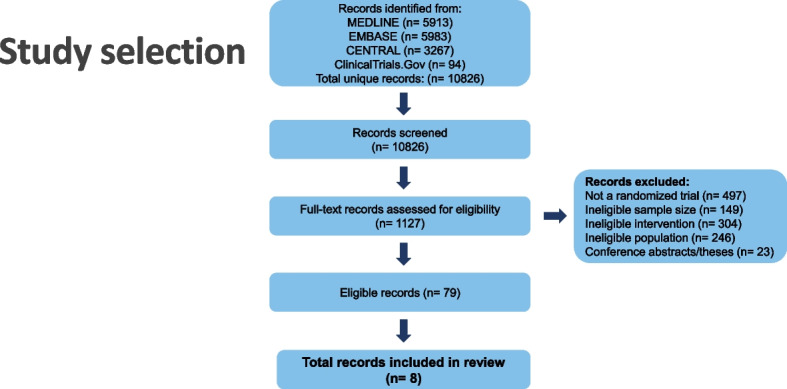
Flow diagram of screened, included, excluded and analyzed studies for the European Headache Federation (EHF) critical re-appraisals and meta-analyses of oral drugs in migraine prevention

Out of 10,826 unique records, eight studies were eligible for at least one outcome analysis, accounting for 2610 patients. The duration of follow-up varied between 16 and 256 weeks. Figure 2 shows the flow diagram of the study selection process. Table 1 summarizes the key characteristics of the included trials.

**Table 1:** Key characteristics of the included trials

Study	Trial name	Interventions vs. placebo	Trial duration (double-blind phase)	Number of randomized patients	Migraine type	MMD at baseline	Mean age (years)	% Male
Silberstein 2004 [30]	MIGR-001	Topiramate 50/100/200 mg	26 weeks	487	EM	6.4	40.3	11.3
Brandes 2004 [6]	MIGR-002	Topiramate 50/100/200 mg	26 weeks	483	EM	6.5	38.8	13.2
Diener 2004 [31]	MIGR-003	Topiramate 100/200 mg Propranolol 160 mg	26 weeks	575	EM	6.0	40.9	20.2
Mei 2004 [32]	-	Topiramate 100 mg	16 weeks	50	EM	5.5	39.2	45.8
Silberstein 2006 [33]	-	Topiramate 200 mg	20 weeks	213	EM	4.9	40.5	14.2
Diener 2007 [34]	TOPMAT-MIG-201	Topiramate 50–200 mg	16 weeks	59	CM	15.9	46.2	25.4
Silberstein 2007 [35] + Silberstein 2009 [36]	Topiramate Chronic Migraine	Topiramate 100 mg	16 weeks	358	CM	15.2	38.2	14.7
Lipton 2011 [37]	INTREPID	Topiramate 100 mg	26 weeks	385	(HF)EM	11.7	40.3	10.9

*CM* Chronic migraine, *EM* Episodic migraine, *HF* High frequency, *MMD* Monthly migraine days. Topiramate doses represent total daily dose

### Narrative description of topiramate in placebo-controlled trials

#### Episodic migraine

In 2004, three MIGR studies (MIGR-001 [30], MIGR-002 [6], and MIGR-003 [31]) were published comparing different doses of topiramate with placebo in patients aged 12–65 years with 3 to 12 migraine attacks per month but no more than 15 monthly headache days. Participants with a maximum of two prior preventive treatments were allowed in the trial, but no concomitant use of another preventive drug was permitted. Primary endpoint was the reduction in mean monthly migraine frequency during the whole 26-week trial period, defined as the number of migraine attacks per month. Monthly migraine days (MMD) and the 50% responder rates were secondary endpoints.

The US-American MIGR-001 study compared three doses of topiramate (50 mg/day, 100 mg/day, 200 mg/day) to placebo [30]. All topiramate doses were associated with higher 50% responder rates compared to placebo (36% for 50 mg, 54% for 100 mg, and 52% for 200 mg vs. 23% for placebo). The two higher topiramate doses led to a significant reduction of MMD (from 6.4 to 3.7 for 100 mg; from 6.1 to 4.0 for 200 mg vs. from 6.1 to 5.2 for placebo), while the 50 mg group did not reach a significant difference. A total of 94 participants discontinued the trial due to adverse events (23% of patients in the topiramate groups vs. 9% of patients in the placebo group). The adverse events that most frequently led to withdrawal comprised of paresthesia, fatigue, nausea, anorexia, and memory and language problems.

The North American MIGR-002 study investigated the same topiramate doses as in the MIGR-001 trial [6]. The 100-mg and 200-mg groups met the primary endpoint, i.e., reduction in the number of monthly migraine attacks vs. placebo. All topiramate groups reached a higher 50% responder rate than placebo (39% for 50 mg, 49% for 100 mg, 47% for 200 mg vs. 23% for placebo). The reduction of MMD also favored topiramate 100 mg (-2.6) and topiramate 200 mg (-2.9) to placebo (-1.3). Withdrawal rates due to adverse events were 17% in the 50-mg group, 27% in the 100-mg group, and 21% in the 200-mg group vs. 12% in the placebo group. The most frequent adverse events leading to withdrawal were paresthesia, fatigue, diarrhea, and cognitive problems.

The multinational MIGR-003 study compared topiramate 100 mg/day or 200 mg/day with placebo and with propranolol 160 mg/day as an active control [31]. The comparison with propranolol is not included in the pooled quantitative analysis of this manuscript. The reduction in the mean number of monthly migraine attacks was significantly different to placebo in the 100-mg group but not in the 200-mg group. Moreover, the 100-mg group was superior to placebo in the reduction of MMD (-1.8 vs. -1.1 for placebo), while both topiramate groups had higher 50% responder rates than placebo (37% for 100 mg, 35% for 200 mg vs. 22% for placebo). Efficacy in the topiramate groups was comparable to propranolol. Adverse events led to treatment discontinuation in 37 patients with topiramate 100 mg (26%), 63 (44%) patients with topiramate 200 mg vs. 15 (10%) patients with placebo, most commonly paresthesia, fatigue, nausea, and cognitive problems.

Mei et al. randomly assigned patients with 2 to 6 migraine attacks per month to receive topiramate or placebo in a 1:1 ratio for 16 weeks [32]. The topiramate start dose was 25 mg/day, which was gradually titrated up to 100 mg/day by the end of the first trial month. The primary endpoint was the reduction of monthly migraine attacks from baseline to weeks 12 to 16. Topiramate was statistically superior to placebo in reaching the primary endpoint (topiramate: from 5.26 to 2.60; placebo: from 5.76 to 4.57). The number of MMD was not reported. In addition, the 50% responder rate was higher in the topiramate group compared to the placebo group (63% vs. 21%). In the topiramate group, 17 patients (29%) discontinued the trial due to adverse events vs. two (4%) in the placebo group. The most common adverse events leading to discontinuation included cognitive problems, paresthesia, weight loss, and somnolence.

Silberstein et al. evaluated the preventive efficacy and tolerability of topiramate 200 mg/day compared to placebo in patients with 3 to 8 migraine attacks per month [33]. Primary endpoint was the change in the mean number of monthly migraine attacks during the entire double-blind study phase. The study did not meet its primary endpoint. Indeed, the reduction in monthly migraine attacks between topiramate 200 mg (-1.42) and placebo (-1.04) was not statistically different. Also, the 50% responder rate was only numerically but not significantly different in the topiramate group compared to placebo (40% vs. 34%). Twenty-one (15%) patients in the topiramate group and four (5%) patients in the placebo group discontinued the trial due to adverse events, including fatigue, nausea, and paresthesia.

The most recent placebo-controlled trial for topiramate is the INTREPID study [37]. The study included high-frequency episodic migraine, defined as 9 to 14 MMD. The aim of the study was to investigate whether topiramate (100 mg/day) can prevent the progression to chronic migraine. Accordingly, the primary endpoint of this trial was the percentage of participants with chronic migraine at month 6. The study revealed similar transformation rates in the topiramate and placebo group and did not meet the primary endpoint. However, topiramate treatment resulted in a significant reduction of MMD compared with placebo (-6.6 vs. -5.3). The 50% responder rates were higher in the topiramate group, but the exact proportions were not reported. Twenty-one (11%) patients in the topiramate group and 18 (9%) patients in the placebo group discontinued the trial due to limiting adverse events. The most common adverse events were paresthesia, fatigue, and dizziness.

#### Chronic migraine

Two studies assessed the preventive properties of topiramate in patients with chronic migraine [34, 35].

The TOPMAT-MIG-201 trial enrolled 59 patients with chronic migraine defined as ≥ 15 MMD for at least three months before trial entry and ≥ 12 MMD during the 4-week baseline phase [34]. Participants were randomized 1:1 to receive either placebo or topiramate in a dose between 50 and 200 mg/day, according to the investigator’s judgement. The study was completed by 38 patients. Primary endpoint was the change in MMD from baseline to the last trial month (-3.5 in the topiramate group vs. + 0.2 in the placebo group). Participants receiving topiramate had significantly higher 50% responder rates compared to placebo (22 vs. 0%). Six (19%) patients in the topiramate group discontinued the study due to insufficient tolerability compared to three (11%) in the placebo group. Overall, the three most common adverse events were paresthesia, nausea, and dizziness.

The second study on chronic migraine by Silberstein et al. randomized 328 participants in a 1:1 ratio to placebo or topiramate with a target dose of 100 mg/day [35]. Overall, 182 subjects continued the study until the end. In this study, the definition of chronic migraine was ≥ 15 monthly headache days, at least 50% of which fulfilled the migraine criteria. The change of monthly migraine/migrainous days from baseline to the entire 16-week double-blind treatment phase was the primary endpoint. A migrainous day was defined as a day with moderate or severe headache with at least one feature among unilaterality, pulsatile character, photophobia and/or photophobia, nausea and/or vomiting, or worsening through physical activity. Treatment with topiramate led to a reduction of -6.4 migraine/migrainous days per month, while placebo reduced them by 4.7 days. The 50% responder rates, published in a subsequent publication, were 37.3% vs. 28.8% for topiramate vs. placebo and differences did not reach statistical significance [36]. In the topiramate group, 18 participants (11%) discontinued the trial due to adverse events, while this was the case for 10 subjects (6%) on placebo. The most frequent adverse events with topiramate were paresthesia, upper respiratory tract infections, and fatigue.

#### Quantitative analysis

All trials described above were included in the quantitative analysis of at least one outcome of interest. Table 2 summarizes key findings of the different meta-analyses.

**Table 2:** Topiramate compared to placebo for migraine prophylaxis

**Patient or population:** migraine **Intervention:** prophylaxis with topiramate **Comparison:** placebo					
**Outcomes**	**№ of participants** **(studies) Follow-up**	**Certainty of the evidence (GRADE)**	**Relative effect (95% CI)**	**Anticipated absolute effects**	
				**Risk with placebo**	**Risk difference with topiramate**
50% or more reduction in monthly migraine days	1,959 (6 RCTs)	**High**	**RR 1.61** (1.29 to 2.01)	275 per 1,000	**168 more per 1,000** (80 more to 278 more)
Monthly migraine days	2,361 (8 RCTs)	**High**	-	N/A	**MD 0.99 migraine days fewer** (1.41 fewer to 0.58 fewer)
Adverse events leading to discontinuation	1000 (8 RCTs)	**High**	**RD 0.08** (0.02 to 0.14)	0 per 1,000	**80 more per 1,000** (20 more to 140 more)

The risk in the intervention group (and its 95% confidence interval) is based on the assumed risk in the comparison group and the relative effect of the intervention (and its 95% CI)

*CI* Confidence interval, *MD* Mean difference, *RCT* Randomized controlled trial, *RR* Risk ratio, *RD* Risk difference

GRADE Working Group grades of evidence

High certainty: we are very confident that the true effect lies close to that of the estimate of the effect

Moderate certainty: we are moderately confident in the effect estimate: the true effect is likely to be close to the estimate of the effect, but there is a possibility that it is substantially different. Low certainty: our confidence in the effect estimate is limited: the true effect may be substantially different from the estimate of the effect

Very low certainty: we have very little confidence in the effect estimate: the true effect is likely to be substantially different from the estimate of the effect

#### Monthly migraine days

**Fig. 3:**
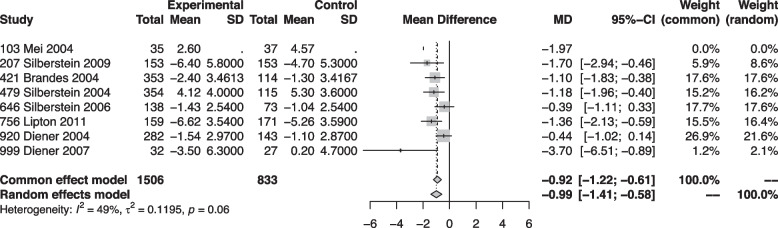
Forest plot showing meta-analysis comparing topiramate with placebo for the reduction of monthly migraine days

Our quantitative analysis for MMD included 2,361 participants from all eight studies (Fig. 3). Six studies provided direct information about MMD [6, 30, 31, 34, 35, 37]. For the remaining two [32, 33], we used the number of monthly migraine attacks as a surrogate parameter.

**Fig. 4:**
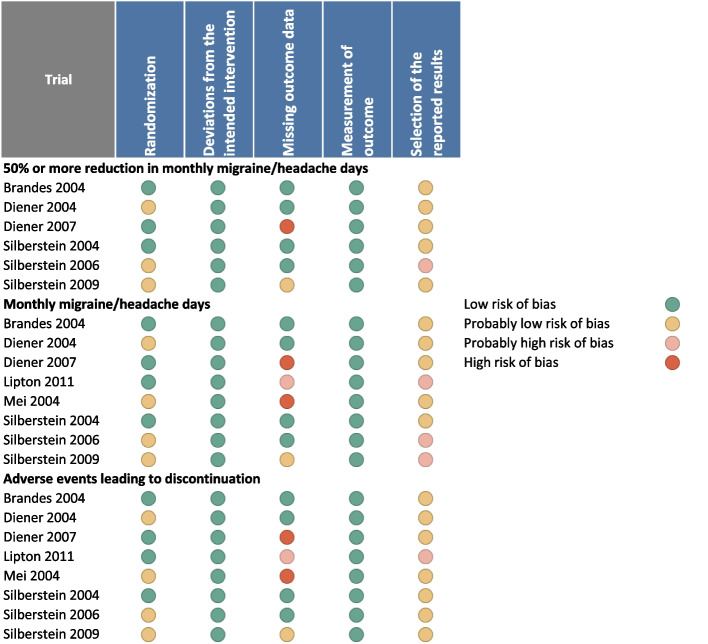
Risk of bias ratings for the randomized controlled trials of topiramate vs. placebo included in this meta-analysis

Overall, the meta-analysis revealed a high certainty evidence that treatment with topiramate reduces migraine frequency over time with a mean difference of 0.99 days compared to placebo. Five studies were deemed at low risk of bias [6, 30, 31, 33, 35], and three studies at high risk of bias [30, 34, 37] (Fig. 4). Reasons for the low rating were mostly missing outcome data.

Pairwise meta-regressions did not show different results for trials at high risk of bias compared with trials at a low risk of bias (Supplementary Material 2). Similarly, a subgroup analysis based on mean MMD at baseline and prior use of migraine preventive treatments revealed similar results as the primary analysis (Supplementary Material 2).

### 50% responder rate

**Fig. 5:**
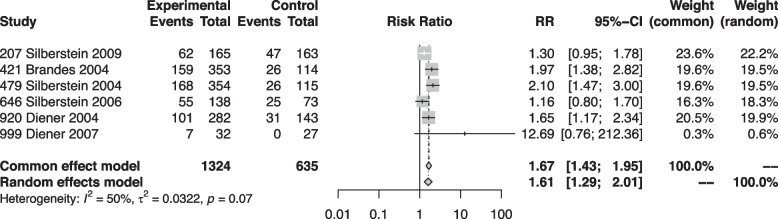
Forest plot showing meta-analysis comparing topiramate with placebo for 50% responder rates

Six trials including 1,959 participants reported the 50% responder rates as an outcome [6, 30, 31, 33, 34, 36]. We found high certainty of evidence that topiramate increased the 50% responder rates (Fig. 5). Across these trials, the relative effect of topiramate compared to placebo was 1.61 (95% CI 1.29–2.01). One trial [34] was rated as high risk of bias and the remaining five as low risk of bias.

Separate subgroup analyses based on baseline migraine frequency, prior use of preventive medication, and risk of bias showed results consistent with the primary analysis (Supplementary Material 3).

### Adverse events leading to discontinuation

**Fig. 6:**
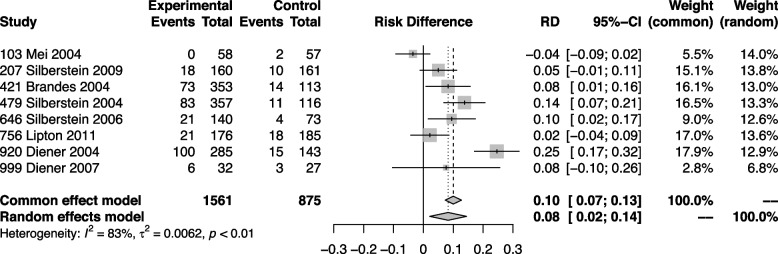
Forest plot showing meta-analysis comparing topiramate with placebo for adverse events leading to discontinuation

Overall, 322 out of 1,561 participants receiving topiramate within eight trials ended the study prematurely due to adverse events [6, 30–35, 37]. Topiramate led to higher discontinuation rates than placebo with a risk difference of 0.08 (95% CI 0.02–0.14) (Fig. 6). The certainty of evidence was deemed as high.

Subgroup analyses based on risk of bias, migraine frequency, and previous preventive medication yielded similar results as the primary analysis (Supplementary Material 4).

## Discussion

In this systematic review and meta-analysis, the existing evidence regarding the efficacy and tolerability of topiramate was revisited, based on data from eight randomized controlled trials (RCTs) with 2,610 individuals with migraine. The certainty of the gathered evidence was judged as high, according to the GRADE system approach. The main findings of this analysis were: 1) patients treated with topiramate showed a 61% higher chance of having a 50% responder rate than patients treated with placebo; 2) the median reduction in the number of MMD was 0.99 migraine day greater than with placebo; and 3) ~ 20% of patients who received topiramate discontinued the treatment due to treatment emergent adverse events.

Topiramate is the only unspecific oral preventive treatment with high certainty evidence for reaching a 50% reduction of MMD [38]. Its efficacy is comparable to other standard oral preventive treatments such as beta-blockers or amitriptyline, but the level of available evidence is higher for topiramate than for the other substances [38]. Of note, it is also the substance with the highest rates of adverse events leading to discontinuation [38].

Tolerability is indeed topiramate’s Achilles tendon. Various adverse events may emerge, but luckily, most of these seem mild and resolve after topiramate discontinuation [6, 30–35, 37]. The most recent version of the Cochrane review, including published evidence until 2013 of topiramate for episodic migraine prophylaxis, showed that adverse events were more frequent in patients treated with topiramate, with a number needed to harm (NNH) ranging from 3 to 25, compared to 2 to 17 in the case of placebo [39]. The adverse events with the lower NNH, in the case of topiramate 100 mg/day, were paresthesia (NNH = 3), taste disturbances (NNH = 14), anorexia (NNH = 17), weight loss (NNH = 17), fatigue (NNH = 25), and memory problems (NNH = 25). In the case of topiramate 200 mg/day, all NNH reduced to approximately half the numbers of 100 mg/day [39]. In addition, congenital malformations and neurodevelopmental disorders, including autism spectrum disorders or intellectual disability in offspring of women exposed to topiramate during pregnancy may occur [40]. According to a recent population-level study, prenatal exposure to topiramate was associated to an adjusted Hazard ratio (aHR) of 1.7 (95% CI, 1.0–2.8) for any neurodevelopmental disorder associated with topiramate doses lower than 100 mg per day and aHR: 2.9 (95%CI, 1.3–6.7) for doses of 100 mg per day or more compared with children from the general population within an 8-year risk period [41]. Given the documented interactions of topiramate beyond the 100 mg/day dosage threshold with oral contraceptives, a cautious approach is warranted when considering topiramate's utilization in women of reproductive age [42]. This led to the European Medicines Agency Pharmacovigilance Risk Assessment Committee towards a recommendation of avoiding topiramate in woman of childbearing age that are not using high efficacy contraceptive methods [43].

In 2004, a study using propranolol as an active comparator showed a higher discontinuation rate due to adverse events for topiramate (26% and 43.7% for topiramate 100 and 200 mg/day respectively) versus 20.3% for propranolol 160 mg/day, while metrics on changes in migraine frequency, 50% responder rate, migraine days, or days of acute medication use were similar within the three groups [31]. In 2019, a parallel group, randomized, open-label study in chronic migraine patients compared topiramate 50–100 mg/day (*n* = 140) versus 155 IE of onabotulinumtoxinA (*n* = 142) [44]. Patients who discontinued topiramate treatment for any reason were allowed to cross over and receive onabotulinumtoxinA. Fewer topiramate-treated patients completed the 32-week period compared to onabotulinumtoxinA (59% vs. 81%). Treatment-emergent adverse events were more frequent in patients treated with topiramate (70% vs. 17%). Efficacy endpoints favored onabotulinumtoxinA [44]. In 2022, a parallel group, randomized, double-blind, double-dummy study (HER-MES) compared topiramate 100 mg/day (*n* = 388) versus erenumab 70–140 mg (*n* = 389) every 4 weeks for a 24-week period [45]. The primary outcome was the proportion of patients with adverse events leading to treatment discontinuation, which was higher in topiramate-treated patients compared with erenumab (38.9% vs. 10.6%). The proportion of patients who achieved a 50% responder rate was lower in the case of topiramate (31.2% vs. 55.4% with erenumab), and patient-reported outcomes were also more favorable in patients treated with erenumab [45].

In the direct comparison between topiramate versus other preventive drugs, the evidence is limited. A parallel group, randomized, double-blind controlled study, conducted between May 2019 and February 2021 compared the change in migraine days per 28 weeks at the end of a 24 weeks treatment period in chronic migraine patients treated with topiramate 100 mg/day (*n* = 46) or propranolol (*n* = 49) [46]. The reduction in migraine days per month was -5.3 ± 1.2 days within patients treated with topiramate, and − 7.3 ± 1.1 days within patients treated with propranolol, albeit differences were not statistically significant [46]. In the case of amitriptyline, a parallel group, randomized, double-blind, double dummy study compared topiramate 100 mg/day (*n* = 172) versus amitriptyline 25 mg/day (*n* = 159), between patients with 3–12 migraine days per month, with no statistically significant differences in the reduction in the number of monthly migraine episodes [47]. Smaller parallel group, randomized, double-blind studies have compared migraine patients treated with topiramate versus amitriptyline (*n* = 73), with no striking differences between groups [48]. In the case of flunarizine, a prospective, open-label study compared the efficacy of 50 mg/day topiramate (*n* = 31) versus 10 mg/day flunarizine (*n* = 31), with better efficacy and tolerability in the flunarizine arm, however, in this study the treatment dose was lower than in most trials, and thus, the results should be interpreted cautiously [49]. This is supported by the results of another open-label prospective study including patients with chronic migraine compared the change in the number of headache days per month during a 12 month period in patients treated with 100 mg/day topiramate (*n* = 44), 5 mg/day flunarizine (*n* = 39) or a combination of topiramate + flunarizine (*n* = 43), with no differences in the efficacy outcomes between groups [50]. Regarding zonisamide, a parallel group, randomized, double blind study compared the efficacy of topiramate 100 mg/day (*n* = 40) versus 200 mg/day zonisamide (*n* = 40) after 12 weeks of treatment, with no differences in the efficacy or tolerability outcomes, except for headache intensity [51].

In the present study, topiramate was only compared to placebo. However, in a recent network meta-analysis that compared calcitonin gene-related peptide (CGRP) monoclonal antibodies (mAbs) and gepants versus oral prophylactic drugs with the same methodology of the present study showed a more favorable profile of the former therapies in terms of efficacy and tolerability [38]. A meta-analysis published in 2021 indirectly compared the efficacy and tolerability of topiramate vs. anti-CGRP mAbs [52]. The efficacy results were relatively similar, expressed as number needed to treat (NNT) to achieve a 50% responder rate (7 vs. 6), while tolerability was far worse in the case of topiramate, with a NNH of 9, compared with 130 in the case of anti-CGRP mAbs [52]. In another comparison to anti-CGRP and onabotulinumtoxinA, with 21 studies accounting for 13,302 patients, topiramate had the highest effect size in the 50% responder rate analysis albeit with the highest drop-out rate as well [53].

One of the main limitations of migraine prophylaxis RCTs is the relatively short duration of the double-blind period, usually 3 or 6 months, while in the real-world setting patients are frequently treated for 9–12 months [54]. The open label extension of one RCT [35] showed that the efficacy of topiramate was sustained during the twelve-months observation period [55]. Moreover, topiramate is the only oral migraine preventive for which sustained efficacy after treatment discontinuation has been documented by an RCT [56]. In 2007, a study included 818 patients that were treated for 26 weeks with topiramate in an open-label phase, followed by a randomized, double-blind, placebo-controlled phase (26 weeks) including 255 patients treated with topiramate (100 mg/day) and 259 patients treated with placebo [56]. The change in the number of migraine days during the last 4 weeks of the open-label phase, compared to the last 4 weeks of the double-blind phase showed a higher difference within patients treated with placebo (+ 1.19 days vs. -0.36 days), but still a sustained benefit compared to the baseline phase. Therefore, these findings suggest that the effect of topiramate may extend beyond the treatment phase. These reasons may explain why topiramate was considered as the preferred drug in the treatment of chronic migraine and the most effective migraine prophylactic drug in a survey including 155 neurologists [57].

The evidence from this systematic review and meta-analysis should be interpreted according to its limitations and the risk of bias of some trials. Additional outcomes, including monthly headache days, number of migraine attacks, acute medication days, patient-reported outcomes and specific adverse events were not characterized. Moreover, migraine attacks were considered as surrogate parameters for monthly migraine days, when these were not available, which might have led to slightly different results in the meta-analysis. In our sensitivity analyses, studies were stratified according to the median baseline migraine frequency, instead on the migraine subtype. This was done because the employed parameter was migraine days and no headache days, however, future studies could evaluate specifically this based on headache days, or according to the migraine subtype. In studies with different dosages of topiramate, the active arms were combined using well-established procedures [24]. Considering only the higher dosages, e.g., by excluding the 50 mg arms, would have led to a better overall efficacy outcome. However, it would have also negatively impacted the estimation about tolerability. Our study differed from prior published systematic reviews and meta-analyses [20] in the criterion of including patients with at least 25 patients per treatment arm, which excluded some studies with smaller sample sizes. Most of the included studies were published previous to the current version of the International Classification of Headache Disorders and diagnostic criteria for chronic migraine have varied. Future studies should identify response and tolerability predictors, and explore how long the benefit lasts, whenever this occurs.

## Conclusion

We found high-certainty evidence in randomized, double-blind, placebo-controlled trials supporting the efficacy of topiramate in the prophylactic treatment of migraine. The proportion of patients on topiramate who achieved a 50% responder rate and the reduction in the number of MMD was higher than in patients treated with placebo. On the other hand, patients treated with topiramate had a higher risk of experiencing a treatment-emergent adverse event leading to treatment discontinuation. The real-world clinical practice of using topiramate is often constrained by its widely recognized low tolerability.

### Supplementary Information

**Additional file 1.** Search Strategies.

**Additional file 2:** **Supplementary Material 2.1.** Subgroup analysis for the reduction in monthly migraine days based on the number of monthly migraine days at baseline (below vs. above the median value). NR = not reported. **Supplementary Material 2.2.** Subgroup analysis for the reduction in monthly migraine days based on the proportion of patients that had previously used prophylactic therapy (below vs. above the median value). NR = not reported. **Supplementary Material 2.3.** Subgroup analysis for the reduction in monthly migraine days based on the risk of bias (high vs. low). Rob = risk of bias.

**Additional file 3: Supplementary Material 3.1.** Subgroup analysis for the 50% responder rate based on the number of monthly migraine days at baseline (below vs. above the median value). NR = not reported. **Supplementary Material 3.2.** Subgroup analysis for the 50% responder rate based on the proportion of patients that had previously used prophylactic therapy (below vs. above the median value). NR = not reported. **Supplementary Material 3.3.** Subgroup analysis for the 50% responder rate based on the risk of bias (high vs. low). Rob = risk of bias.

**Additional file 4: Supplementary Material 4.1.** Subgroup analysis for adverse events leading to discontinuation based on the number of monthly migraine days at baseline (below vs. above the median value). NR = not reported. **Supplementary Material 4.2.** Subgroup analysis for adverse events leading to discontinuation based on the proportion of patients that had previously used prophylactic therapy (below vs. above the median value). NR = not reported. **Supplementary Material 4.3.** Subgroup analysis for adverse events leading to discontinuation based on the risk of bias (high vs. low). Rob = risk of bias.

## Data Availability

All data generated or analysed during this study are included in this published article [and its supplementary information files].

## Abbreviations

AMPA: α-Amino-3-hydroxy-5-methyl-4-isoxazolepropionic acid
CGRP: Calcitonin Gene-Related Peptide
CI: Confidence Interval
CM: Chronic Migraine
EM: Episodic Migraine
GABA: γ-AminoButyric Acid
HFEM: High Frequency Episodic Migraine
MMD: Monthly Migraine Days
NNH: Number Needed to Harm
NNT: Number Needed to Treat
NR: Not Reported
PRISMA: Preferred Reporting Items for Systematic Reviews
RCT: Randomized Controlled Trial
RD: Risk Difference
rob: Risk Of Bias
RR: Risk Ratio
