# Supplementary material for: European Headache Federation (EHF) critical reappraisal and meta-analysis of oral drugs in migraine prevention – part 3: topiramate
Source: J Headache Pain. 2023 Oct 10;24(1):134. doi: 10.1186/s10194-023-01671-5 (PMC10563338; doi:10.1186/s10194-023-01671-5)
Supplement: Supplementary file 1 — Additional file 1. Search Strategies. [file 10194_2023_1671_MOESM1_ESM.pdf]

Search Strategies

Topic:

The comparative effectiveness of CGRP monoclonal antibody therapies and other pharmacotherapies for migraine prevention: a protocol for a systematic review and network meta-analysis.

PRISMA Initial Results: total number of results before duplicates removed

**Database [Platform]** Searches run August 13, 2022. *No date, language limits used.*	**Results**
OVID Medline Epub Ahead of Print, In-Process & Other Non-Indexed Citations, Ovid MEDLINE(R) Daily and Ovid MEDLINE(R) 1946 to Present [Ovid]	5,913
Embase 1974 to 2022 August 12 [Ovid]	5,983
EBM Reviews - Cochrane Central Register of Controlled Trials July 2022 [Ovid]	3,267
**TOTAL**	**15,163**

ClinicalTrials.gov Results Searched August 13, 2022.

Studies With Results | Interventional Studies | Migraine Disorders | various treatment terms [see strategies below in document]	94
**TOTAL**	**94**

ClinicalTrials.gov Search Strategies

1 Study found for: CGRP monoclonal antibodies | Studies With Results | Interventional Studies | Migraine Disorders

8 Studies found for: adrenergic beta-antagonists | Studies With Results | Interventional Studies | Migraine Disorders

1 Study found for: Calcium Channel Blocker | Studies With Results | Interventional Studies | Migraine Disorders

22 Studies found for: Anticonvulsants | Studies With Results | Interventional Studies | Migraine Disorders

39 Studies found for: Anti-Inflammatory Agents, Non-Steroidal | Studies With Results | Interventional Studies | Migraine Disorders

1 Study found for: angiotensin receptor antagonists | Studies With Results | Interventional Studies | Migraine Disorders

5 Studies found for: antidepressive agents | Studies With Results | Interventional Studies | Migraine Disorders

11 Studies found for: Botulinum Toxins, Type A | Studies With Results | Interventional Studies | Migraine Disorders

No Studies found for: coenzyme Q10 | Studies With Results | Interventional Studies | Migraine Disorders

3 Studies found for: Magnesium | Studies With Results | Interventional Studies | Migraine Disorders

3 Studies found for: Melatonin | Studies With Results | Interventional Studies | Migraine Disorders

MEDLINE(R) 1996 to August 12, 2022
Search Strategy:

#	Searches	Results
1	exp Migraine Disorders/	22110
2	(migraine or migraines or sick headache or sick headaches or hemicrania or migrainous headache or status hemicranicus).tw,kf.	28623
3	or/1-2	30802

4	("cgrp monoclonal antibodies" or "calcitonin gene-related peptide antibodies (cgrp) monoclonal antibodies (mabs)" or "cgrp mabs").tw,kf.	127
5	calcitonin gene related peptide.tw,kf.	8169
6	Calcitonin Gene-Related Peptide Receptor Antagonists/	848
7	exp Antibodies, Monoclonal/	191819
8	6 and 7	274
9	adrenergic beta-antagonists/	24650
10	("adrenergic beta antagonist*" or "adrenergic beta blocker*" or "adrenergic beta receptor blockader*" or "beta adrenergic antagonist*" or "beta adrenergic blocker*" or "beta adrenergic blocking agent*" or "beta adrenergic blocking drug" or "beta adrenergic receptor antagonist*" or "beta adrenergic receptor blockader*" or "beta adrenergic receptor blocker*" or "beta adrenoceptor antagonist*" or "beta adrenoceptor blocker*" or "beta adrenoceptor blocking agent*" or "beta adrenoceptor blocking drug*" or "beta adrenolytic" or "beta antagonist*" or "beta antiadrenergic agent*" or "beta blocker" or "beta blocking adrenergic agent*" or "beta blocking agent*" or "beta blocking drug*" or "beta receptor adrenergic blocking agent*" or "beta receptor blocker*" or "beta receptor blocking agent*" or "beta sympathicolytic agent*" or "beta sympathicolytics" or "beta sympatholytic agent*" or "betasympatholytic agent*").tw,kf.	14206
11	alprenolol/ or brimonidine tartrate, timolol maleate drug combination/ or bunolol/ or bupranolol/ or carteolol/ or carvedilol/ or dihydroalprenolol/ or iodocyanopindolol/ or labetalol/ or levobunolol/ or metipranolol/ or nadolol/ or oxprenolol/ or penbutolol/ or pindolol/ or propranolol/ or sotalol/ or timolol/	14502
12	(alprenolol or bucindolol or bunolol or bupranolol or carazolol or carpindolol or carteolol or carvedilol or cloranolol or dexpropranolol or dichlorisoprenaline or dihydroalprenolol or dilevalol or iodocyanopindolol or isamoltane or labetalol or levobunolol or metipranolol or nadolol or nifenalol or oxprenolol or penbutolol or pindolol or propranolol or sotalol or spirendolol or tertatolol or tilisolol or timolol).tw,kf.	19291
13	adrenergic beta-1 receptor antagonists/	1281
14	acebutolol/ or atenolol/ or betaxolol/ or bisoprolol/ or celiprolol/ or metoprolol/ or practolol/	6735
15	(acebutolol or atenolol or bendacalol or betaxolol or bevantolol or bisoprolol or celiprolol or cetamolol or cyanoiodopindolol or cyanopindolol or dramedilol or epanolol	22643

	or esmolol or flusoxolol or landiolol or metoprolol or nebivolol or practolol or propranolol or ritodrine or salcardolol or sandoz 204545 or sotalol or talinolol or vortioxetine).tw,kf.	
16	Calcium Channel Blockers/	25268
17	("calcium blocker" or "calcium blocking agent*" or "calcium channel antagonist*" or "calcium channel blocker*" or "calcium channel blocking drug*" or "calcium entry blocker" or "calcium entry blocking agent*" or "calcium inhibitor" or "exogenous calcium antagonist*" or "exogenous calcium blockader*").tw,kf.	12479
18	Anticonvulsants/	40862
19	("anti convulsant agent*" or "anti convulsive agent*" or "anti convulsive drug*" or "anti epileptic*" or "anticonvulsant*" or "anticonvulsive agent*" or "anticonvulsive drug*" or "anticonvulsivum" or "antiepileptic" or "antiepileptics" or "antiepileptiform drug*").tw,kf.	34720
20	acetazolamide/ or bromides/ or cannabidiol/ or carbamazepine/ or chlormethiazole/ or clobazam/ or clonazepam/ or clorazepate dipotassium/ or diazepam/ or dimethadione/ or estazolam/ or ethosuximide/ or felbamate/ or flunarizine/ or gabapentin/ or lacosamide/ or lamotrigine/ or levetiracetam/ or lorazepam/ or magnesium sulfate/ or medazepam/ or mephenytoin/ or mephobarbital/ or meprobamate/ or nitrazepam/ or oxcarbazepine/ or paraldehyde/ or phenobarbital/ or phenytoin/ or pregabalin/ or primidone/ or riluzole/ or thiopental/ or tiagabine/ or tiletamine/ or topiramate/ or trimethadione/ or valproic acid/ or vigabatrin/ or zonisamide/	57455
21	(acetazolamide or albutoin or alprazolam or ascorbate magnesium or belnacasan or brexanolone or brivaracetam or bromides or cannabidiol or cannabidivarin or carabersat or carbamazepine or carisbamate or cenobamate or chlormethiazole or clobazam or clomethiazole or clonazepam or clorazepate dipotassium or darigabat or dextromethorphan or dezinamide or diazepam or diclofenamide or dimethadione or elpetrigine or estazolam or eterobarb or ethosuximide or ethotoin or etiracetam or felbamate or fenfluramine or flunarizine or gabapentin or ganaxolone or guaifenesin or "ica 105665" or imepitoin or imidazenil or lacosamide or lamotrigine or lanicemine or levetiracetam or licarbazepine or lorazepam or loreclezole or losigamone or magnesium sulfate or medazepam or mephenytoin or mephobarbital or meprobamate or mesuximide or metharbital or midazolam or nitrazepam or oxcarbazepine or padsevonil or paraldehyde or paramethadione or pentoxyverine or perampanel or phenobarbital or phenytoin or pregabalin or primidone or progabide or remacemide or retigabine or riluzole or ropizine or rufinamide or safinamide or seletracetam or sodium bromide or	82787

	stiripentol or sultiame or talampanel or thiopental or tiagabine or tiletamine or topiramate or trimethadione or valproic acid or valpromide or valrocemide or vigabatrin or vixotrigine or zaleplon or zonisamide).tw,kf.	
22	exp Anti-Inflammatory Agents, Non-Steroidal/	138244
23	("anti inflammatory analgesic*" or "aspirin like agent*" or "non steroid antiinflammatory agent*" or "non steroid antiinflammatory drug*" or "non steroidal anti inflammatory agent*" or "non steroidal anti inflammatory drug*" or "non steroidal antiinflammatory agent*" or "non steroidal antiinflammatory drug*" or "nonsteroid antiinflammatory agent*" or "nonsteroid antiinflammatory drug" or "nonsteroid antirheumatic agent" or "nonsteroidal anti inflammatory agent*" or "nonsteroidal anti inflammatory drug*" or "nonsteroidal antiinflammatory agent*" or "nonsteroidal antiinflammatory drug*" or "nsaid" or "nsaids").tw,kf.	36916
24	Acetaminophen/ or Antipyrine/ or Aspirin/ or Celecoxib/ or Clonixin/ or Curcumin/ or Diclofenac/ or dipyrone/ or Ibuprofen/ or Indomethacin/ or Ketoprofen/ or Ketorolac/ or Ketorolac Tromethamine/ or Mesalamine/ or Naproxen/ or salicylates/ or Sulfasalazine/ or Tolmetin/	96692
25	(acalabrutinib or aceclofenac or acemetacin or acetaminophen or acetaminosalol or acetylsalicylic acid or actarit or adalimumab or alemtuzumab or antipyrine or apremilast or ascriptin or aspirin or azathioprine or azelaic acid or balsalazide or belimumab or brimonidine or celecoxib or clonixin lysine or clonixin or curcumin or dexibuprofen or dexketoprofen or diclofenac or dipyrone or etodolac or etoricoxib or fenoprofen or flurbiprofen or ibuprofen or icosapentaenoic acid or indometacin or indomethacin or ketoprofen or ketorolac or leflunomide or lornoxicam or loxoprofen or lumiracoxib or meclofenamic acid or mefenamic acid or meloxicam or mesalamine or mesalazine or methotrexate or nabumetone or naproxen or natalizumab or nimesulide or parecoxib or phenylbutazone or piroxicam or pirprofen or rasagiline or rituximab or rofecoxib or ruxolitinib or salazosulfapyridine or salicylates or salicylic acid or satralizumab or sulfasalazine or sulindac or tenoxicam or teriflunomide or tofacitinib or tolmetin or valdecoxib).tw,kf.	203782
26	exp Angiotensin Receptor Antagonists/	24152
27	("angiotensin ii receptor antagonist*" or "angiotensin ii receptor blocker*" or "angiotensin ii receptor blocking agent*" or "angiotensin receptor antagonist" or "angiotensin receptor blocker*" or "angiotensin receptor blocking agent*").tw,kf.	12160
28	irbesartan/ or olmesartan medoxomil/ or telmisartan/ or valsartan/	6612

29	("angiotensin 1 receptor antagonist" or "angiotensin 2 receptor antagonist" or azilsartan or candesartan or eprosartan or fimasartan or irbesartan or losartan or olmesartan or sartan derivative or tasosartan or telmisartan or trv027 or valsartan).tw,kf.	17916
30	exp Antidepressive Agents/	94901
31	("anti depressant agent*" or "antidepressant*" or "antidepression drug*" or "antidepressive agent*" or "antidepressive drug*" or "neurothymoleptic agent*" or "psychoenergizer" or "thymoanaleptic" or "thymoanaleptics" or thymoleptic or thymoleptics or "thymolytic agent").tw,kf.	52365
32	Aripiprazole/ or Duloxetine Hydrochloride/ or Lithium Carbonate/ or Lithium Compounds/ or Mirtazapine/ or Moclobemide/ or Phenelzine/ or Pizotyline/ or Quetiapine Fumarate/ or Sertraline/ or Tranylcypromine/	16745
33	Bupropion/ or Citalopram/ or Fluoxetine/ or Mianserin/ or Paroxetine/ or Sulpiride/ or Trazodone/ or Tryptophan/ or Venlafaxine Hydrochloride/	38437
34	Amitriptyline/ or Clomipramine/ or Desipramine/ or Dothiepin/ or Doxepin/ or Imipramine/ or Nortriptyline/ or Opipramol/	7959
35	(agomelatine or amitriptyline or aprepitant or aripiprazole or asenapine or "botulinum toxin a" or bupropion or citalopram or clomipramine or desipramine or dothiepin or doxepin or duloxetine hydrochloride or esketamine or fluoxetine or gepirone or imipramine or indalpine or ipsapirone or lithium acetate or lithium carbonate or lithium chloride or lithium compounds or lithium salt or mianserin or mifepristone or minaprine or mirtazapine or moclobemide or monoamine oxidase inhibitor* or noradrenalin uptake inhibitor* or nortriptyline or opipramol or paroxetine or phenelzine or pizotyline or quetiapine fumarate or serotonin uptake inhibitor* or sertraline or sulpiride or tetracyclic antidepressant* or tranylcypromine or trazodone or tricyclic antidepressant* or tryptophan or venlafaxine hydrochloride).tw,kf.	84436
36	Botulinum Toxins, Type A/	10631
37	("abobotulinum toxin a" or "abobotulinumtoxin a" or "abobotulinumtoxina" or "agn 151607" or "agn151607" or "alluzience" or "ant 1207" or "ant 1401" or "ant 1403" or "ant1207" or "ant1401" or "ant1403" or "azzalure" or "bocouture" or "boe−tox" or "bont a" or "bont serotype a" or "botox" or "botulin a" or "botulin toxin a" or "botulinium a toxin" or "botulinum a exotoxin" or "botulinum a toxin" or "botulinum neurotoxin a" or "botulinum toxin a" or "btxa" or "clostridium botulinum a toxin" or "clostridium botulinum endotoxin" or "clostridium botulinum neurotoxin a" or	7610

	"clostridium botulinum toxin type a" or "clostridium botulinum type a neurotoxin" or "cnt 52120" or "cnt52120" or "daxibotulinum toxin a" or "daxibotulinumtoxin a" or "daxibotulinumtoxina" or "dwp 450" or "dwp450" or "dyslor" or "dysport" or "evabotulinum toxin a" or "evabotulinumtoxin a" or "evabotulinumtoxina" or "evosyal" or "gemibotulinum toxin a" or "gemibotulinumtoxin a" or "gemibotulinumtoxina" or "gsk 1358820" or "gsk1358820" or "incobotulinum toxin a" or "incobotulinumtoxin a" or "incobotulinumtoxina" or "ipn 59011" or "ipn59011" or "jeuveau" or "letibotulinum toxin a" or "letibotulinumtoxin a" or "letibotulinumtoxina" or "meditoxin" or "mt 10109" or "mt10109" or "nabota" or "neuronox" or "nivobotulinum toxin a" or "nivobotulinumtoxin a" or "nivobotulinumtoxina" or "nt 201" or "nt201" or "nuceiva" or "oculinum" or "onabotulinum toxin a" or "onabotulinumtoxin a" or "onabotulinumtoxina" or "onaclostox" or "pm 12759" or "pm12759" or "prabotulinum toxin a" or "prabotulinumtoxin a" or "prabotulinumtoxina" or "prosigne" or "purtox" or "qm 1114" or "qm1114" or "relabotulinum toxin a" or "relabotulinumtoxin a" or "relabotulinumtoxina" or "reloxin" or "rtt 150" or "rtt150" or "vistabel" or "vistabex" or "xeomeen" or "xeomin").tw,kf.	
38	(caomet or "coenzyme 910" or "coenzyme q 10" or "coenzyme Q10" or decorenone or mitocor or neuquinone or "quinone q 10" or ubidecarenone or ubimaior or "ubiquinone (10)" or "ubiquinone 10" or "ubiquinone 50" or ubiten).tw,kf.	4064
39	Magnesium/	24624
40	(magnesium or romag).tw,kf.	32593
41	Melatonin/	17282
42	("apl 510" or "apl510" or ceyesto or circadin or "jan 13004" or "jan13004" or "ki 1001" or "ki1001" or melatonin or melatonina or melovine or orlogin or slenyto or "sp 13004" or "sp13004" or waferest).tw,kf.	20405
43	or/4-5,8-42	696356
44	3 and 43 [migraines AND prophylaxis]	6793
45	randomized controlled trial.pt.	480050
46	controlled clinical trial.pt.	49673
47	randomized.ab.	460234
48	placebo.ab.	169835
49	drug therapy.fs.	1880993
50	randomly.ab.	294766
51	trial.ab.	482925

52	groups.ab.	1714791
53	45 or 46 or 47 or 48 or 49 or 50 or 51 or 52	3949647
54	animals/ not humans.sh.	2817192
55	53 not 54	3422601
56	44 and 55 [migraines AND prophylaxis AND RCTs]	4566
57	remove duplicates from 56	4540

OVID Medline Epub Ahead of Print, In-Process & Other Non-Indexed Citations, Ovid MEDLINE(R) Daily and Ovid MEDLINE(R) 1946 to Present
Search Strategy:

#	Searches	Results
1	exp Migraine Disorders/	30669
2	(migraine or migraines or sick headache or sick headaches or hemicrania or migrainous headache or status hemicranicus).tw,kf.	40663
3	or/1-2	44880
4	("cgrp monoclonal antibodies" or "calcitonin gene-related peptide antibodies (cgrp) monoclonal antibodies (mabs)" or "cgrp mabs").tw,kf.	167
5	calcitonin gene related peptide.tw,kf.	12349
6	Calcitonin Gene-Related Peptide Receptor Antagonists/	869
7	exp Antibodies, Monoclonal/	267646
8	6 and 7	274
9	adrenergic beta-antagonists/	41510
10	("adrenergic beta antagonist*" or "adrenergic beta blocker*" or "adrenergic beta receptor blockader*" or "beta adrenergic antagonist*" or "beta adrenergic blocker*" or "beta adrenergic blocking agent*" or "beta adrenergic blocking drug" or "beta adrenergic receptor antagonist*" or "beta adrenergic receptor blockader*" or "beta adrenergic receptor blocker*" or "beta adrenoceptor antagonist*" or "beta adrenoceptor blocker*" or "beta adrenoceptor blocking agent*" or "beta adrenoceptor blocking drug*" or "beta adrenolytic" or "beta antagonist*" or "beta antiadrenergic agent*" or "beta blocker" or "beta blocking adrenergic agent*" or "beta blocking agent*" or "beta blocking drug*" or "beta receptor adrenergic blocking agent*" or "beta receptor blocker*" or "beta receptor blocking agent*" or "beta sympathicolytic agent*"	28363

	or "beta sympathicolytics" or "beta sympatholytic agent*" or "betasympatholytic agent*").tw,kf.	
11	alprenolol/ or brimonidine tartrate, timolol maleate drug combination/ or bunolol/ or bupranolol/ or carteolol/ or carvedilol/ or dihydroalprenolol/ or iodocyanopindolol/ or labetalol/ or levobunolol/ or metipranolol/ or nadolol/ or oxprenolol/ or penbutolol/ or pindolol/ or propranolol/ or sotalol/ or timolol/	48975
12	(alprenolol or bucindolol or bunolol or bupranolol or carazolol or carpindolol or carteolol or carvedilol or cloranolol or dexpropranolol or dichlorisoprenaline or dihydroalprenolol or dilevalol or iodocyanopindolol or isamoltane or labetalol or levobunolol or metipranolol or nadolol or nifenalol or oxprenolol or penbutolol or pindolol or propranolol or sotalol or spirendolol or tertatolol or tilisolol or timolol).tw,kf.	51527
13	adrenergic beta-1 receptor antagonists/	1328
14	acebutolol/ or atenolol/ or betaxolol/ or bisoprolol/ or celiprolol/ or metoprolol/ or practolol/	14374
15	(acebutolol or atenolol or bendacalol or betaxolol or bevantolol or bisoprolol or celiprolol or cetamolol or cyanoiodopindolol or cyanopindolol or dramedilol or epanolol or esmolol or flusoxolol or landiolol or metoprolol or nebivolol or practolol or propranolol or ritodrine or salcardolol or sandoz 204545 or sotalol or talinolol or vortioxetine).tw,kf.	54958
16	Calcium Channel Blockers/	37478
17	("calcium blocker" or "calcium blocking agent*" or "calcium channel antagonist*" or "calcium channel blocker*" or "calcium channel blocking drug*" or "calcium entry blocker" or "calcium entry blocking agent*" or "calcium inhibitor" or "exogenous calcium antagonist*" or "exogenous calcium blockader*").tw,kf.	20506
18	Anticonvulsants/	54979
19	("anti convulsant agent*" or "anti convulsive agent*" or "anti convulsive drug*" or "anti epileptic*" or "anticonvulsant*" or "anticonvulsive agent*" or "anticonvulsive drug*" or "anticonvulsivum" or "antiepileptic" or "antiepileptics" or "antiepileptiform drug*").tw,kf.	52702
20	acetazolamide/ or bromides/ or cannabidiol/ or carbamazepine/ or chlormethiazole/ or clobazam/ or clonazepam/ or clorazepate dipotassium/ or diazepam/ or dimethadione/ or estazolam/ or ethosuximide/ or felbamate/ or flunarizine/ or gabapentin/ or lacosamide/ or lamotrigine/ or levetiracetam/ or lorazepam/ or magnesium sulfate/ or	121063

	medazepam/ or mephenytoin/ or mephobarbital/ or meprobamate/ or nitrazepam/ or oxcarbazepine/ or paraldehyde/ or phenobarbital/ or phenytoin/ or pregabalin/ or primidone/ or riluzole/ or thiopental/ or tiagabine/ or tiletamine/ or topiramate/ or trimethadione/ or valproic acid/ or vigabatrin/ or zonisamide/	
21	(acetazolamide or albutoin or alprazolam or ascorbate magnesium or belnacasan or brexanolone or brivaracetam or bromides or cannabidiol or cannabidivarin or carabersat or carbamazepine or carisbamate or cenobamate or chlormethiazole or clobazam or clomethiazole or clonazepam or clorazepate dipotassium or darigabat or dextromethorphan or dezinamide or diazepam or diclofenamide or dimethadione or elpetrigine or estazolam or eterobarb or ethosuximide or ethotoin or etiracetam or felbamate or fenfluramine or flunarizine or gabapentin or ganaxolone or guaifenesin or "ica 105665" or imepitoin or imidazenil or lacosamide or lamotrigine or lanicemine or levetiracetam or licarbazepine or lorazepam or loreclezole or losigamone or magnesium sulfate or medazepam or mephenytoin or mephobarbital or meprobamate or mesuximide or metharbital or midazolam or nitrazepam or oxcarbazepine or padsevonil or paraldehyde or paramethadione or pentoxyverine or perampanel or phenobarbital or phenytoin or pregabalin or primidone or progabide or remacemide or retigabine or riluzole or ropizine or rufinamide or safinamide or seletracetam or sodium bromide or stiripentol or sultiame or talampanel or thiopental or tiagabine or tiletamine or topiramate or trimethadione or valproic acid or valpromide or valrocemide or vigabatrin or vixotrigine or zaleplon or zonisamide).tw,kf.	148034
22	exp Anti-Inflammatory Agents, Non-Steroidal/	211076
23	("anti inflammatory analgesic*" or "aspirin like agent*" or "non steroid antiinflammatory agent*" or "non steroid antiinflammatory drug*" or "non steroidal anti inflammatory agent*" or "non steroidal anti inflammatory drug*" or "non steroidal antiinflammatory agent*" or "non steroidal antiinflammatory drug*" or "nonsteroid antiinflammatory agent*" or "nonsteroid antiinflammatory drug" or "nonsteroid antirheumatic agent" or "nonsteroidal anti inflammatory agent*" or "nonsteroidal anti inflammatory drug*" or "nonsteroidal antiinflammatory agent*" or "nonsteroidal antiinflammatory drug*" or "nsaid" or "nsaids").tw,kf.	51073
24	Acetaminophen/ or Antipyrine/ or Aspirin/ or Celecoxib/ or Clonixin/ or Curcumin/ or Diclofenac/ or dipyrone/ or Ibuprofen/ or Indomethacin/ or Ketoprofen/ or Ketorolac/ or Ketorolac Tromethamine/ or Mesalamine/ or Naproxen/ or salicylates/ or Sulfasalazine/ or Tolmetin/	156025

25	(acalabrutinib or aceclofenac or acemetacin or acetaminophen or acetaminosalol or acetylsalicylic acid or actarit or adalimumab or alemtuzumab or antipyrine or apremilast or ascriptin or aspirin or azathioprine or azelaic acid or balsalazide or belimumab or brimonidine or celecoxib or clonixin lysine or clonixin or curcumin or dexibuprofen or dexketoprofen or diclofenac or dipyrone or etodolac or etoricoxib or fenoprofen or flurbiprofen or ibuprofen or icosapentaenoic acid or indometacin or indomethacin or ketoprofen or ketorolac or leflunomide or lornoxicam or loxoprofen or lumiracoxib or meclofenamic acid or mefenamic acid or meloxicam or mesalamine or mesalazine or methotrexate or nabumetone or naproxen or natalizumab or nimesulide or parecoxib or phenylbutazone or piroxicam or pirprofen or rasagiline or rituximab or rofecoxib or ruxolitinib or salazosulfapyridine or salicylates or salicylic acid or satralizumab or sulfasalazine or sulindac or tenoxicam or teriflunomide or tofacitinib or tolmetin or valdecoxib).tw,kf.	307676
26	exp Angiotensin Receptor Antagonists/	26926
27	("angiotensin ii receptor antagonist*" or "angiotensin ii receptor blocker*" or "angiotensin ii receptor blocking agent*" or "angiotensin receptor antagonist" or "angiotensin receptor blocker*" or "angiotensin receptor blocking agent*").tw,kf.	14264
28	irbesartan/ or olmesartan medoxomil/ or telmisartan/ or valsartan/	6658
29	("angiotensin 1 receptor antagonist" or "angiotensin 2 receptor antagonist" or azilsartan or candesartan or eprosartan or fimasartan or irbesartan or losartan or olmesartan or sartan derivative or tasosartan or telmisartan or trv027 or valsartan).tw,kf.	20660
30	exp Antidepressive Agents/	157396
31	("anti depressant agent*" or "antidepressant*" or "antidepression drug*" or "antidepressive agent*" or "antidepressive drug*" or "neurothymoleptic agent*" or "psychoenergizer" or "thymoanaleptic" or "thymoanaleptics" or thymoleptic or thymoleptics or "thymolytic agent").tw,kf.	73764
32	Aripiprazole/ or Duloxetine Hydrochloride/ or Lithium Carbonate/ or Lithium Compounds/ or Mirtazapine/ or Moclobemide/ or Phenelzine/ or Pizotyline/ or Quetiapine Fumarate/ or Sertraline/ or Tranylcypromine/	21662
33	Bupropion/ or Citalopram/ or Fluoxetine/ or Mianserin/ or Paroxetine/ or Sulpiride/ or Trazodone/ or Tryptophan/ or Venlafaxine Hydrochloride/	61997
34	Amitriptyline/ or Clomipramine/ or Desipramine/ or Dothiepin/ or Doxepin/ or Imipramine/ or Nortriptyline/ or Opipramol/	24431

35	(agomelatine or amitriptyline or aprepitant or aripiprazole or asenapine or "botulinum toxin a" or bupropion or citalopram or clomipramine or desipramine or dothiepin or doxepin or duloxetine hydrochloride or esketamine or fluoxetine or gepirone or imipramine or indalpine or ipsapirone or lithium acetate or lithium carbonate or lithium chloride or lithium compounds or lithium salt or mianserin or mifepristone or minaprine or mirtazapine or moclobemide or monoamine oxidase inhibitor* or noradrenalin uptake inhibitor* or nortriptyline or opipramol or paroxetine or phenelzine or pizotyline or quetiapine fumarate or serotonin uptake inhibitor* or sertraline or sulpiride or tetracyclic antidepressant* or tranylcypromine or trazodone or tricyclic antidepressant* or tryptophan or venlafaxine hydrochloride).tw,kf.	143344
36	Botulinum Toxins, Type A/	10662
37	("abobotulinum toxin a" or "abobotulinumtoxin a" or "abobotulinumtoxina" or "agn 151607" or "agn151607" or "alluzience" or "ant 1207" or "ant 1401" or "ant 1403" or "ant1207" or "ant1401" or "ant1403" or "azzalure" or "bocouture" or "boe−tox" or "bont a" or "bont serotype a" or "botox" or "botulin a" or "botulin toxin a" or "botulinium a toxin" or "botulinum a exotoxin" or "botulinum a toxin" or "botulinum neurotoxin a" or "botulinum toxin a" or "btxa" or "clostridium botulinum a toxin" or "clostridium botulinum endotoxin" or "clostridium botulinum neurotoxin a" or "clostridium botulinum toxin type a" or "clostridium botulinum type a neurotoxin" or "cnt 52120" or "cnt52120" or "daxibotulinum toxin a" or "daxibotulinumtoxin a" or "daxibotulinumtoxina" or "dwp 450" or "dwp450" or "dyslor" or "dysport" or "evabotulinum toxin a" or "evabotulinumtoxin a" or "evabotulinumtoxina" or "evosyal" or "gemibotulinum toxin a" or "gemibotulinumtoxin a" or "gemibotulinumtoxina" or "gsk 1358820" or "gsk1358820" or "incobotulinum toxin a" or "incobotulinumtoxin a" or "incobotulinumtoxina" or "ipn 59011" or "ipn59011" or "jeuveau" or "letibotulinum toxin a" or "letibotulinumtoxin a" or "letibotulinumtoxina" or "meditoxin" or "mt 10109" or "mt10109" or "nabota" or "neuronox" or "nivobotulinum toxin a" or "nivobotulinumtoxin a" or "nivobotulinumtoxina" or "nt 201" or "nt201" or "nuceiva" or "oculinum" or "onabotulinum toxin a" or "onabotulinumtoxin a" or "onabotulinumtoxina" or "onaclostox" or "pm 12759" or "pm12759" or "prabotulinum toxin a" or "prabotulinumtoxin a" or "prabotulinumtoxina" or "prosigne" or "purtox" or "qm 1114" or "qm1114" or "relabotulinum toxin a" or "relabotulinumtoxin a" or "relabotulinumtoxina" or "reloxin" or "rtt 150" or "rtt150" or "vistabel" or "vistabex" or "xeomeen" or "xeomin").tw,kf.	9189

38	(caomet or "coenzyme 910" or "coenzyme q 10" or "coenzyme Q10" or decorenone or mitocor or neuquinone or "quinone q 10" or ubidecarenone or ubimaior or "ubiquinone (10)" or "ubiquinone 10" or "ubiquinone 50" or ubiten).tw,kf.	5376
39	Magnesium/	69283
40	(magnesium or romag).tw,kf.	66468
41	Melatonin/	22186
42	("apl 510" or "apl510" or ceyesto or circadin or "jan 13004" or "jan13004" or "ki 1001" or "ki1001" or melatonin or melatonina or melovine or orlogin or slenyto or "sp 13004" or "sp13004" or waferest).tw,kf.	28086
43	or/4-5,8-42	1183314
44	3 and 43 [migraines AND prophylaxis]	9301
45	randomized controlled trial.pt.	574929
46	controlled clinical trial.pt.	94985
47	randomized.ab.	571967
48	placebo.ab.	230792
49	drug therapy.fs.	2519979
50	randomly.ab.	389035
51	trial.ab.	612187
52	groups.ab.	2393254
53	45 or 46 or 47 or 48 or 49 or 50 or 51 or 52	5433260
54	animals/ not humans.sh.	5002581
55	53 not 54	4733125
56	44 and 55 [migraines AND prophylaxis AND RCTs]	5941
57	remove duplicates from 56	5913

EBM Reviews - Cochrane Central Register of Controlled Trials July 2022
Search Strategy:

#	Searches	Results
1	exp Migraine Disorders/	3020
2	(migraine or migraines or sick headache or sick headaches or hemicrania or migrainous headache or status hemicranicus).tw,kw.	9024
3	1 or 2	9137

4	("cgrp monoclonal antibodies" or "calcitonin gene-related peptide antibodies (cgrp) monoclonal antibodies (mabs)" or "cgrp mabs").tw,kw.	13
5	calcitonin gene related peptide.tw,kw.	1062
6	Calcitonin Gene-Related Peptide Receptor Antagonists/	81
7	exp Antibodies, Monoclonal/	15809
8	6 and 7	42
9	adrenergic beta-antagonists/	4417
10	("adrenergic beta antagonist*" or "adrenergic beta blocker*" or "adrenergic beta receptor blockader*" or "beta adrenergic antagonist*" or "beta adrenergic blocker*" or "beta adrenergic blocking agent*" or "beta adrenergic blocking drug" or "beta adrenergic receptor antagonist*" or "beta adrenergic receptor blockader*" or "beta adrenergic receptor blocker*" or "beta adrenoceptor antagonist*" or "beta adrenoceptor blocker*" or "beta adrenoceptor blocking agent*" or "beta adrenoceptor blocking drug*" or "beta adrenolytic" or "beta antagonist*" or "beta antiadrenergic agent*" or "beta blocker" or "beta blocking adrenergic agent*" or "beta blocking agent*" or "beta blocking drug*" or "beta receptor adrenergic blocking agent*" or "beta receptor blocker*" or "beta receptor blocking agent*" or "beta sympathicolytic agent*" or "beta sympathicolytics" or "beta sympatholytic agent*" or "betasympatholytic agent*").tw,kw.	6202
11	alprenolol/ or "brimonidine tartrate, timolol maleate drug combination"/ or bunolol/ or bupranolol/ or carteolol/ or carvedilol/ or dihydroalprenolol/ or iodocyanopindolol/ or labetalol/ or levobunolol/ or metipranolol/ or nadolol/ or oxprenolol/ or penbutolol/ or pindolol/ or propranolol/ or sotalol/ or timolol/	6240
12	(alprenolol or bucindolol or bunolol or bupranolol or carazolol or carpindolol or carteolol or carvedilol or cloranolol or dexpropranolol or dichlorisoprenaline or dihydroalprenolol or dilevalol or iodocyanopindolol or isamoltane or labetalol or levobunolol or metipranolol or nadolol or nifenalol or oxprenolol or penbutolol or pindolol or propranolol or sotalol or spirendolol or tertatolol or tilisolol or timolol).tw,kw.	10888
13	adrenergic beta-1 receptor antagonists/	215
14	acebutolol/ or atenolol/ or betaxolol/ or bisoprolol/ or celiprolol/ or metoprolol/ or practolol/	4201
15	(acebutolol or atenolol or betaxolol or bevantolol or bisoprolol or celiprolol or cetamolol or cyanoiodopindolol or cyanopindolol or esmolol or landiolol or metoprolol or nebivolol or practolol or propranolol or ritodrine or sotalol or talinolol or vortioxetine).tw,kw.	13586
16	Calcium Channel Blockers/	2876

17	("calcium blocker" or "calcium blocking agent*" or "calcium channel antagonist*" or "calcium channel blocker*" or "calcium channel blocking drug*" or "calcium entry blocker" or "calcium entry blocking agent*" or "calcium inhibitor" or "exogenous calcium antagonist*" or "exogenous calcium blockader*").tw,kw.	3325
18	Anticonvulsants/	2440
19	("anti convulsant agent*" or "anti convulsive agent*" or "anti convulsive drug*" or "anti epileptic*" or "anticonvulsant*" or "anticonvulsive agent*" or "anticonvulsive drug*" or "anticonvulsivum" or "antiepileptic" or "antiepileptics" or "antiepileptiform drug*").tw,kw.	4495
20	acetazolamide/ or bromides/ or cannabidiol/ or carbamazepine/ or chlormethiazole/ or clobazam/ or clonazepam/ or clorazepate dipotassium/ or diazepam/ or dimethadione/ or estazolam/ or ethosuximide/ or felbamate/ or flunarizine/ or gabapentin/ or lacosamide/ or lamotrigine/ or levetiracetam/ or lorazepam/ or magnesium sulfate/ or medazepam/ or mephenytoin/ or mephobarbital/ or meprobamate/ or nitrazepam/ or oxcarbazepine/ or paraldehyde/ or phenobarbital/ or phenytoin/ or pregabalin/ or primidone/ or riluzole/ or thiopental/ or tiagabine/ or tiletamine/ or topiramate/ or trimethadione/ or valproic acid/ or vigabatrin/ or zonisamide/	11619
21	(acetazolamide or albutoin or alprazolam or ascorbate magnesium or belnacasan or brexanolone or brivaracetam or bromides or cannabidiol or cannabidivarin or carabersat or carbamazepine or carisbamate or cenobamate or chlormethiazole or clobazam or clomethiazole or clonazepam or clorazepate dipotassium or darigabat or dextromethorphan or dezinamide or diazepam or diclofenamide or dimethadione or elpetrigine or estazolam or eterobarb or ethosuximide or ethotoin or etiracetam or felbamate or fenfluramine or flunarizine or gabapentin or ganaxolone or guaifenesin or "ica 105665" or imepitoin or imidazenil or lacosamide or lamotrigine or lanicemine or levetiracetam or licarbazepine or lorazepam or loreclezole or losigamone or magnesium sulfate or medazepam or mephenytoin or mephobarbital or meprobamate or mesuximide or metharbital or midazolam or nitrazepam or oxcarbazepine or padsevonil or paraldehyde or paramethadione or pentoxyverine or perampanel or phenobarbital or phenytoin or pregabalin or primidone or progabide or remacemide or retigabine or riluzole or ropizine or rufinamide or safinamide or seletracetam or sodium bromide or stiripentol or sultiame or talampanel or thiopental or tiagabine or tiletamine or topiramate or trimethadione or valproic acid or valpromide or valrocemide or vigabatrin or vixotrigine or zaleplon or zonisamide).tw,kw.	34246
22	exp Anti-Inflammatory Agents, Non-Steroidal/	21883

23	("anti inflammatory analgesic*" or "aspirin like agent*" or "non steroid antiinflammatory agent*" or "non steroid antiinflammatory drug*" or "non steroidal anti inflammatory agent*" or "non steroidal anti inflammatory drug*" or "non steroidal antiinflammatory agent*" or "non steroidal antiinflammatory drug*" or "nonsteroid antiinflammatory agent*" or "nonsteroid antiinflammatory drug" or "nonsteroid antirheumatic agent" or "nonsteroidal anti inflammatory agent*" or "nonsteroidal anti inflammatory drug*" or "nonsteroidal antiinflammatory agent*" or "nonsteroidal antiinflammatory drug*" or "nsaid" or "nsaids").tw,kw.	11038
24	Acetaminophen/ or Antipyrine/ or Aspirin/ or Celecoxib/ or Clonixin/ or Curcumin/ or Diclofenac/ or dipyrone/ or Ibuprofen/ or Indomethacin/ or Ketoprofen/ or Ketorolac/ or Ketorolac Tromethamine/ or Mesalamine/ or Naproxen/ or salicylates/ or Sulfasalazine/ or Tolmetin/	19158
25	(acalabrutinib or aceclofenac or acemetacin or acetaminophen or acetaminosalol or acetylsalicylic acid or actarit or adalimumab or alemtuzumab or antipyrine or apremilast or ascriptin or aspirin or azathioprine or azelaic acid or balsalazide or belimumab or brimonidine or celecoxib or clonixin lysine or clonixin or curcumin or dexibuprofen or dexketoprofen or diclofenac or dipyrone or etodolac or etoricoxib or fenoprofen or flurbiprofen or ibuprofen or icosapentaenoic acid or indometacin or indomethacin or ketoprofen or ketorolac or leflunomide or lornoxicam or loxoprofen or lumiracoxib or meclofenamic acid or mefenamic acid or meloxicam or mesalamine or mesalazine or methotrexate or nabumetone or naproxen or natalizumab or nimesulide or parecoxib or phenylbutazone or piroxicam or pirprofen or rasagiline or rituximab or rofecoxib or ruxolitinib or salazosulfapyridine or salicylates or salicylic acid or satralizumab or sulfasalazine or sulindac or tenoxicam or teriflunomide or tofacitinib or tolmetin or valdecoxib).tw,kw.	70589
26	exp angiotensin receptor antagonists/	4178
27	("angiotensin ii receptor antagonist*" or "angiotensin ii receptor blocker*" or "angiotensin ii receptor blocking agent*" or "angiotensin receptor antagonist" or "angiotensin receptor blocker*" or "angiotensin receptor blocking agent*").tw,kw.	3582
28	irbesartan/ or olmesartan medoxomil/ or telmisartan/ or valsartan/	2080
29	("angiotensin 1 receptor antagonist" or "angiotensin 2 receptor antagonist" or azilsartan or candesartan or eprosartan or fimasartan or irbesartan or losartan or olmesartan or sartan derivative or tasosartan or telmisartan or trv027 or valsartan).tw,kw.	8620
30	exp antidepressive agents/	15881

31	("anti depressant agent*" or "antidepressant*" or "antidepression drug*" or "antidepressive agent*" or "antidepressive drug*" or "neurothymoleptic agent*" or "psychoenergizer" or "thymoanaleptic" or "thymoanaleptics" or thymoleptic or thymoleptics or "thymolytic agent").tw,kw.	13906
32	Aripiprazole/ or Duloxetine Hydrochloride/ or Lithium Carbonate/ or Lithium Compounds/ or Mirtazapine/ or Moclobemide/ or Phenelzine/ or Pizotyline/ or Quetiapine Fumarate/ or Sertraline/ or Tranylcypromine/	4101
33	Bupropion/ or Citalopram/ or Fluoxetine/ or Mianserin/ or Paroxetine/ or Sulpiride/ or Trazodone/ or Tryptophan/ or Venlafaxine Hydrochloride/	6608
34	Amitriptyline/ or Clomipramine/ or Desipramine/ or Dothiepin/ or Doxepin/ or Imipramine/ or Nortriptyline/ or Opipramol/	3410
35	(agomelatine or amitriptyline or aprepitant or aripiprazole or asenapine or "botulinum toxin a" or bupropion or citalopram or clomipramine or desipramine or dothiepin or doxepin or duloxetine hydrochloride or esketamine or fluoxetine or gepirone or imipramine or indalpine or ipsapirone or lithium acetate or lithium carbonate or lithium chloride or lithium compounds or lithium salt or mianserin or mifepristone or minaprine or mirtazapine or moclobemide or monoamine oxidase inhibitor* or noradrenalin uptake inhibitor* or nortriptyline or opipramol or paroxetine or phenelzine or pizotyline or quetiapine fumarate or serotonin uptake inhibitor* or sertraline or sulpiride or tetracyclic antidepressant* or tranylcypromine or trazodone or tricyclic antidepressant* or tryptophan or venlafaxine hydrochloride).tw,kw.	25811
36	Botulinum Toxins, Type A/	1835
37	("abobotulinum toxin a" or "abobotulinumtoxin a" or "abobotulinumtoxina" or "agn 151607" or "agn151607" or "alluzience" or "ant 1207" or "ant 1401" or "ant 1403" or "ant1207" or "ant1401" or "ant1403" or "azzalure" or "bocouture" or "boe−tox" or "bont a" or "bont serotype a" or "botox" or "botulin a" or "botulin toxin a" or "botulinium a toxin" or "botulinum a exotoxin" or "botulinum a toxin" or "botulinum neurotoxin a" or "botulinum toxin a" or "btxa" or "clostridium botulinum a toxin" or "clostridium botulinum endotoxin" or "clostridium botulinum neurotoxin a" or "clostridium botulinum toxin type a" or "clostridium botulinum type a neurotoxin" or "cnt 52120" or "cnt52120" or "daxibotulinum toxin a" or "daxibotulinumtoxin a" or "daxibotulinumtoxina" or "dwp 450" or "dwp450" or "dyslor" or "dysport" or "evabotulinum toxin a" or "evabotulinumtoxin a" or "evabotulinumtoxina" or "evosyal" or "gemibotulinum toxin a" or "gemibotulinumtoxin a" or "gemibotulinumtoxina" or "gsk 1358820" or "gsk1358820" or "incobotulinum toxin a" or "incobotulinumtoxin a" or "incobotulinumtoxina" or "ipn	3672

	59011" or "ipn59011" or "jeuveau" or "letibotulinum toxin a" or "letibotulinumtoxin a" or "letibotulinumtoxina" or "meditoxin" or "mt 10109" or "mt10109" or "nabota" or "neuronox" or "nivobotulinum toxin a" or "nivobotulinumtoxin a" or "nivobotulinumtoxina" or "nt 201" or "nt201" or "nuceiva" or "oculinum" or "onabotulinum toxin a" or "onabotulinumtoxin a" or "onabotulinumtoxina" or "onaclostox" or "pm 12759" or "pm12759" or "prabotulinum toxin a" or "prabotulinumtoxin a" or "prabotulinumtoxina" or "prosigne" or "purtox" or "qm 1114" or "qm1114" or "relabotulinum toxin a" or "relabotulinumtoxin a" or "relabotulinumtoxina" or "reloxin" or "rtt 150" or "rtt150" or "vistabel" or "vistabex" or "xeomeen" or "xeomin").tw,kw.	
38	(caomet or "coenzyme 910" or "coenzyme q 10" or "coenzyme Q10" or decorenone or mitocor or neuquinone or "quinone q 10" or ubidecarenone or ubimaior or "ubiquinone (10)" or "ubiquinone 10" or "ubiquinone 50" or ubiten).tw,kw.	1080
39	Magnesium/	1229
40	(magnesium or romag).tw,kw.	7739
41	Melatonin/	1328
42	("apl 510" or "apl510" or ceyesto or circadin or "jan 13004" or "jan13004" or "ki 1001" or "ki1001" or melatonin or melatonina or melovine or orlogin or slenyto or "sp 13004" or "sp13004" or waferest).tw,kw.	3246
43	or/4-5,8-42	197886
44	3 and 43	3267
