# Supplementary material for: European Headache Federation (EHF) critical reappraisal and meta-analysis of oral drugs in migraine prevention – part 3: topiramate
Source: J Headache Pain. 2023 Oct 10;24(1):134. doi: 10.1186/s10194-023-01671-5 (PMC10563338; doi:10.1186/s10194-023-01671-5)
Supplement: Supplementary file 2 — Additional file 2: Supplementary Material 2.1. Subgroup analysis for the reduction in monthly migraine days based on the number of monthly migraine days at baseline (below vs. above the median value). NR = not reported. Supplementary Material 2.2. Subgroup analysis for the reduction in monthly migraine days based on the proportion of patients that had previously used prophylactic therapy (below vs. above the median value). NR = not reported. Supplementary Material 2.3. Subgroup analysis for the reduction in monthly migraine days based on the risk of bias (high vs. low). Rob = risk of bias. [file 10194_2023_1671_MOESM2_ESM.pdf]

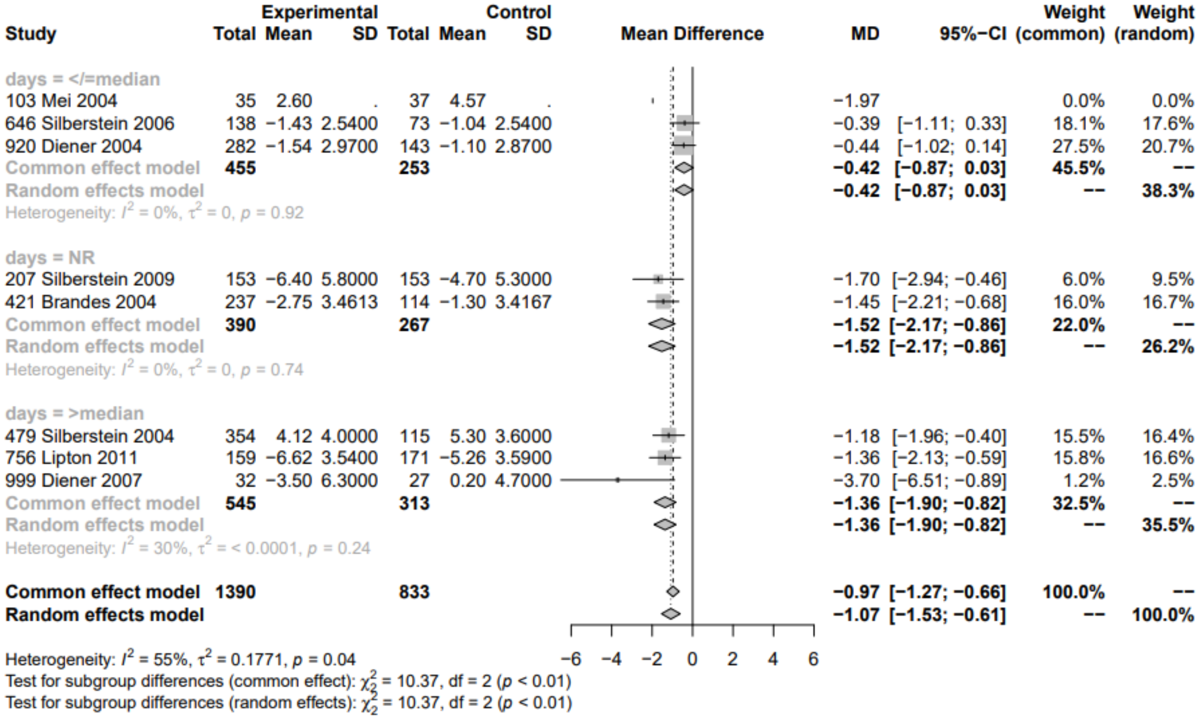

Supplementary Material 2.1: Subgroup analysis for the reduction in monthly migraine days based on the number of monthly migraine days at baseline (below vs. above the median value). NR = not reported.

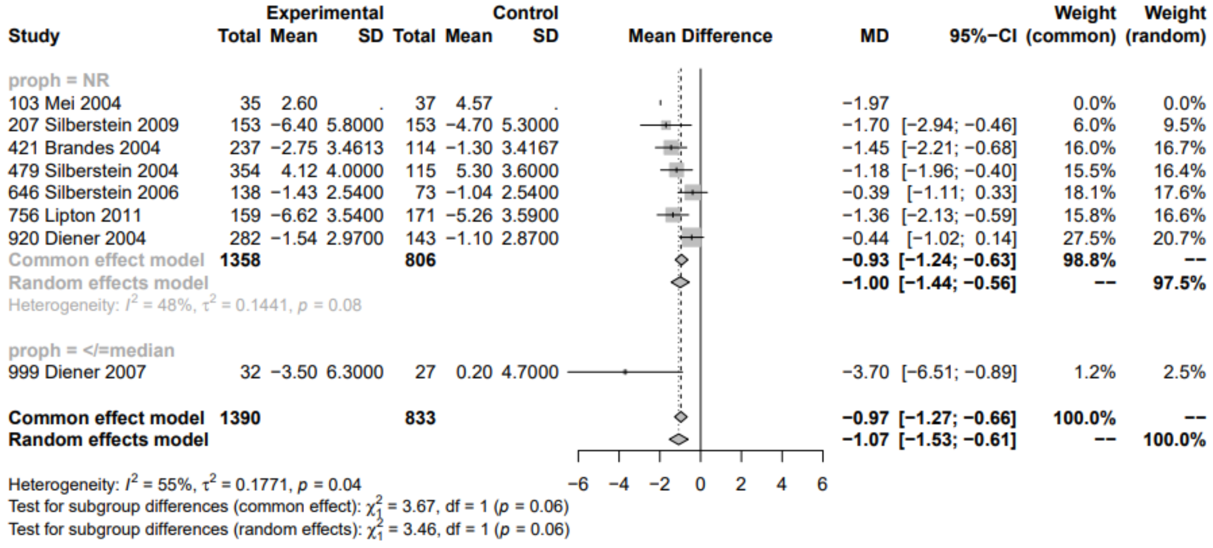

Supplementary Material 2.2: Subgroup analysis for the reduction in monthly migraine days based on the proportion of patients that had previously used prophylactic therapy (below vs. above the median value). NR = not reported.

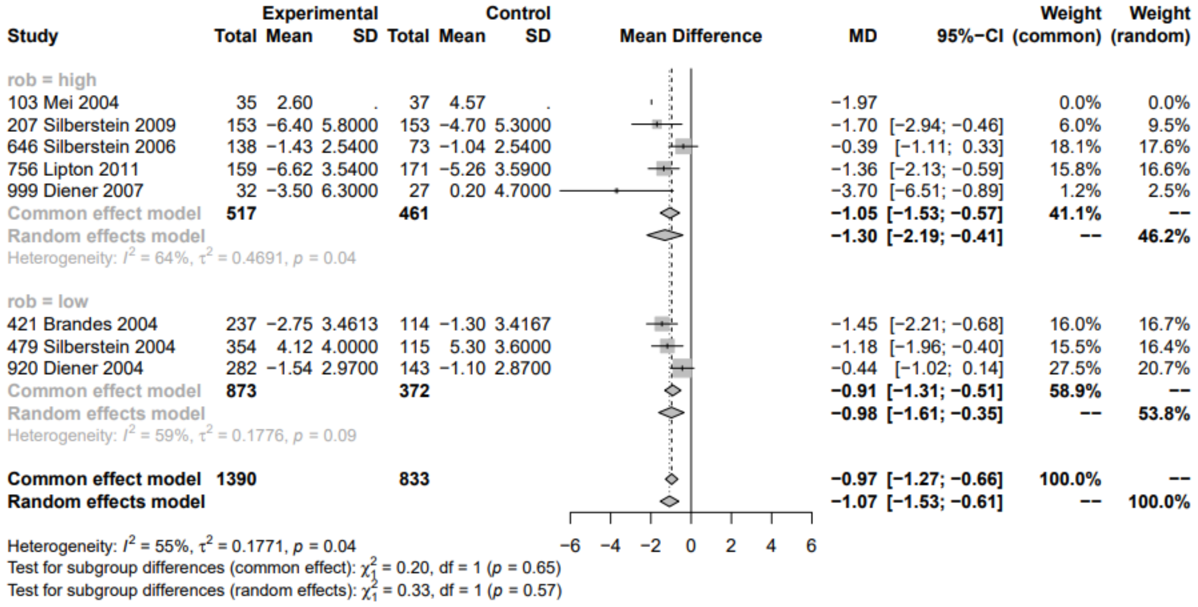

Supplementary Material 2.3: Subgroup analysis for the reduction in monthly migraine days based on the risk of bias (high vs. low). Rob = risk of bias.
