# Supplementary material for: European Headache Federation (EHF) critical reappraisal and meta-analysis of oral drugs in migraine prevention – part 3: topiramate
Source: J Headache Pain. 2023 Oct 10;24(1):134. doi: 10.1186/s10194-023-01671-5 (PMC10563338; doi:10.1186/s10194-023-01671-5)
Supplement: Supplementary file 3 — Additional file 3: Supplementary Material 3.1. Subgroup analysis for the 50% responder rate based on the number of monthly migraine days at baseline (below vs. above the median value). NR = not reported. Supplementary Material 3.2. Subgroup analysis for the 50% responder rate based on the proportion of patients that had previously used prophylactic therapy (below vs. above the median value). NR = not reported. Supplementary Material 3.3. Subgroup analysis for the 50% responder rate based on the risk of bias (high vs. low). Rob = risk of bias. [file 10194_2023_1671_MOESM3_ESM.pdf]

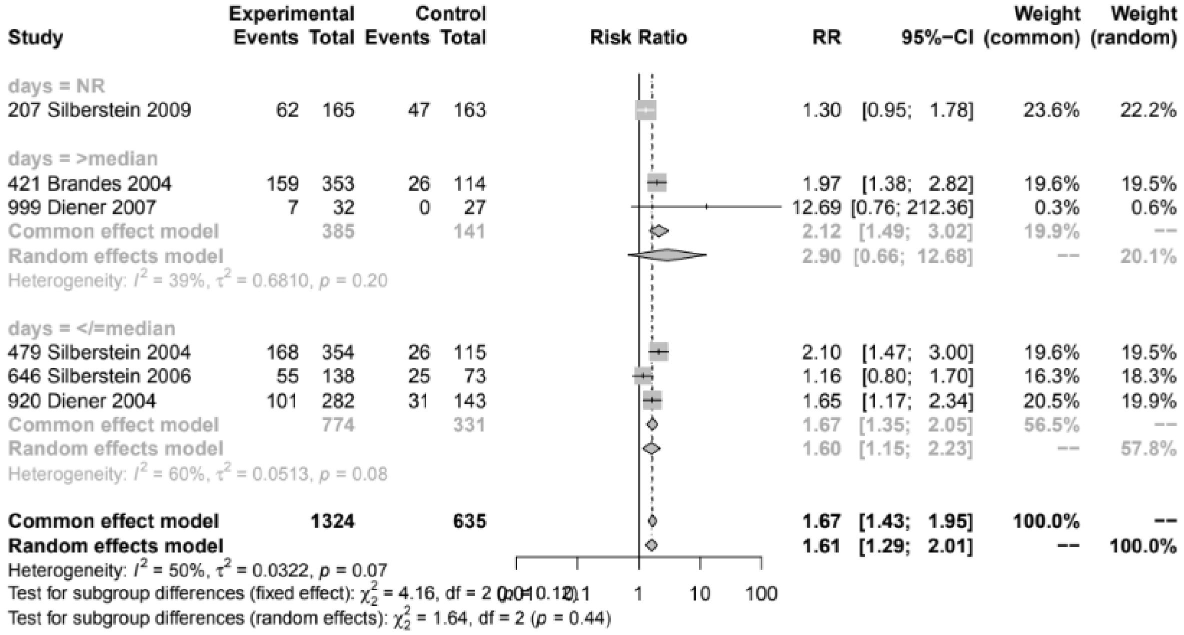

Supplementary Material 3.1: Subgroup analysis for the 50% responder rate based on the number of monthly migraine days at baseline (below vs. above the median value). NR = not reported.

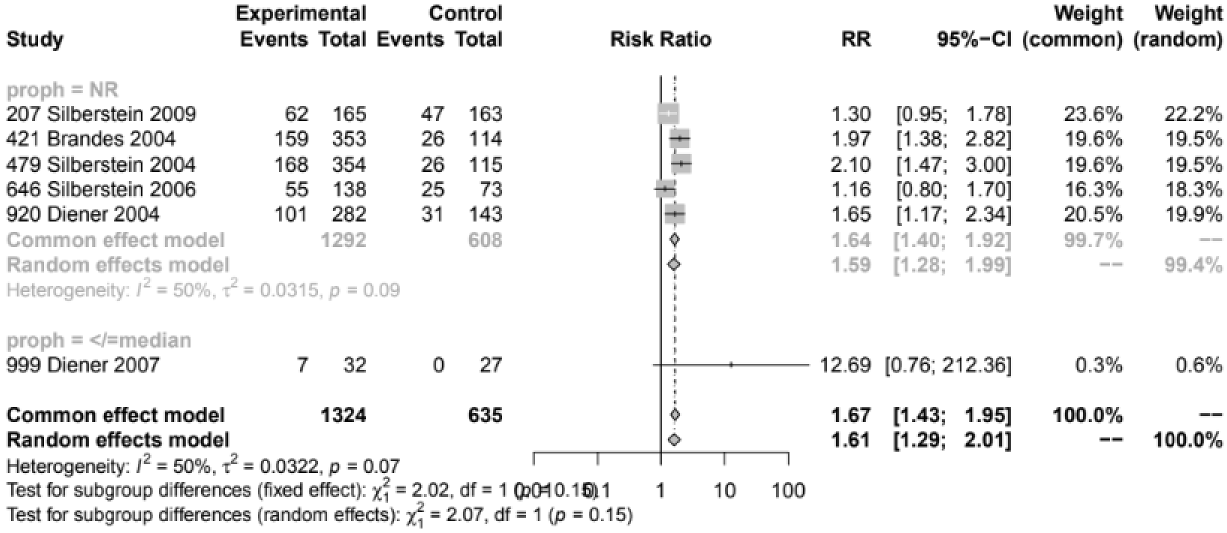

Supplementary Material 3.2: Subgroup analysis for the 50% responder rate based on the proportion of patients that had previously used prophylactic therapy (below vs. above the median value). NR = not reported.

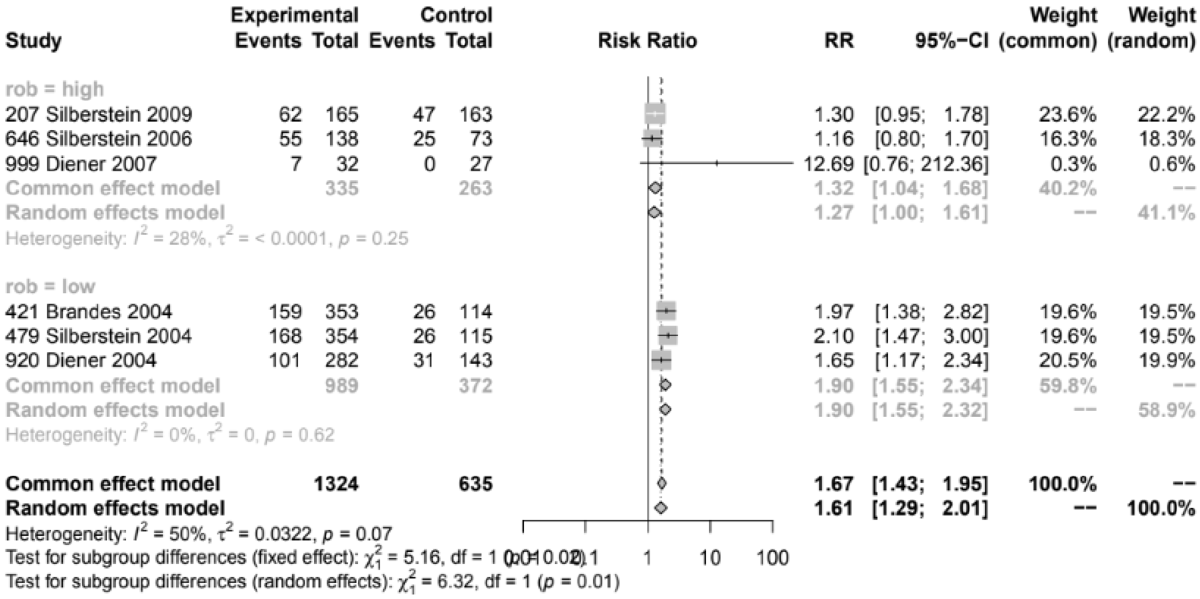

Supplementary Material 3.3: Subgroup analysis for the 50% responder rate based on the risk of bias (high vs. low). Rob = risk of bias.
